# Oligomerization of protein arginine methyltransferase 1 and its functional impact on substrate arginine methylation

**DOI:** 10.1016/j.jbc.2024.107947

**Published:** 2024-11-02

**Authors:** Tran Dang, Nadendla EswarKumar, Sunil Kumar Tripathi, Chunli Yan, Chun-Hsiung Wang, Mengtong Cao, Tanmoy Kumar Paul, Elizabeth Oladoyin Agboluaje, May P. Xiong, Ivaylo Ivanov, Meng-Chiao Ho, Y. George Zheng

**Affiliations:** 1 Department of Pharmaceutical and Biomedical Sciences, College of Pharmacy, University of Georgia, Athens, Georgia, United States; 2 Institute of Biological Chemistry, Academia Sinica, Taipei, Taiwan; 3 Department of Chemistry, Georgia State University, Atlanta, Georgia, USA; 4 Center for Diagnostics and Therapeutics, Georgia State University, Atlanta, Georgia, USA; 5 Institute of Biochemical Sciences, National Taiwan University, Taipei, Taiwan; 6 Graduate Institute of Biochemistry and Molecular Biology, National Taiwan University, Taipei, Taiwan

**Keywords:** PRMT1, protein arginine methyltransferase, arginine methylation, oligomerization, cryo-EM, protein structure, enzyme activity regulation, molecular modeling

## Abstract

Protein arginine methyltransferases (PRMTs) are important posttranslational modifying enzymes in eukaryotic proteins and regulate diverse pathways from gene transcription, RNA splicing, and signal transduction to metabolism. Increasing evidence supports that PRMTs exhibit the capacity to form higher-order oligomeric structures, but the structural basis of PRMT oligomerization and its functional consequence are elusive. Herein, we revealed for the first time different oligomeric structural forms of the predominant arginine methyltransferase PRMT1 using cryo-EM, which included tetramer (dimer of dimers), hexamer (trimer of dimers), octamer (tetramer of dimers), decamer (pentamer of dimers), and also helical filaments. Through a host of biochemical assays, we showed that PRMT1 methyltransferase activity was substantially enhanced as a result of the high-ordered oligomerization. High-ordered oligomerization increased the catalytic turnover and the multimethylation processivity of PRMT1. Presence of a catalytically dead PRMT1 mutant also enhanced the activity of WT PRMT1, pointing out a noncatalytic role of oligomerization. Structural modeling demonstrates that oligomerization enhances substrate retention at the PRMT1 surface through electrostatic force. Our studies offered key insights into PRMT1 oligomerization and established that oligomerization constitutes a novel molecular mechanism that positively regulates the enzymatic activity of PRMTs in biology.

Protein arginine methylation is a versatile posttranslational modification mark that is found to play regulatory roles in assorted cellular processes in eukaryotic cells, especially gene transcription, RNA processing, and signal transduction (1, 2, 3, 4). The family of protein arginine methyltransferases (PRMTs) consists of nine enzyme members, each classified as type-I, II, or III enzyme, depending on the nature of the methylated arginine products generated by the catalytic process (5, 6, 7, 8). All PRMTs are capable of producing monomethylarginine by transferring the methyl group from the methyl donor, SAM, to the guanidino nitrogen of arginine. Type I PRMTs can further catalyze the transfer of a second methyl group onto the same ω-nitrogen atom to create asymmetric dimethylarginines. On the contrary, type II PRMTs catalyze transferring the second methyl group on to the different terminal ω-nitrogen atom to form symmetric dimethylarginine. Type III PRMT, with PRMT7 being the only member, catalyzes the formation of monomethylarginine but is unable to catalyze the second methylation event. The dysregulation of PRMTs is of great biomedical significance for a variety of diseases (5, 9, 10). In oncology, PRMTs have been evidenced to associate with all stages of cancer development: initiation, progression, and metastasis (11, 12). While many studies have been conducted elaborating on phenotypical manifestations of PRMT changes in health and disease, there is a clear demand for structural and biochemical investigations to uncover how PRMTs are regulated at the molecular level. Such information is especially critical to aid the design of small molecule drugs targeting PRMTs.

It is well established that the homodimerization of PRMTs is an absolute requirement for their catalytic activity (13, 14, 15). The homodimeric architecture creates a bowl-like ring shape where the dimerization arm of one subunit interacts with the opposite subunit of the dimer. The diminishing of the dimerization arm of PRMT1 depletes the methylation activity of PRMT1 (16). Except for the dimerization, a few studies have pointed that PRMTs, including PRMT1, are capable of oligomerizing to form higher order homo-oligomeric architectures (14, 17, 18). The X-ray crystal structure of yeast *Saccharomyces cerevisiae* PRMT1 (Hmt1) demonstrates a hexameric structure (a trimer of dimers) (19), although such a structure has not been found in any PRMTs in other species. By chemical crosslinking, we previously found that PRMT1 exists in different oligomer forms and oligomerization is concentration-dependent and biochemical analysis showed that PRMT1 activity increases as a result of the oligomerization, though the mechanism is unknown (20). These conclusions about PRMT1 oligomerization was independently proved later by Toma-Fukai et al. (21). The Hevel group showed PRMT1 predominantly exists as a tetramer using native gel electrophoresis and analytical ultracentrifugation, supporting the oligomerization process of PRMT1 (15, 22). Our previously resolved X-ray crystallographic structure of PRMT8 shows that it exists as a tetramer form (a dimer of dimers) and the β15 strand plays a vital role in the assembly of the tetramer (23). The tetramer form of PRMT8 was also confirmed in size exclusion chromatography (SEC) and analytical ultracentrifugation studies. Interestingly, an independent study showed that human PRMT8 can form octamers and even helical filaments (21). Despite these studies, there have been no structural information to date that holistically address different forms of PRMT oligomerization and no clear mechanistic understanding has emerged of PRMT oligomerization and its downstream function.

This work is to investigate mechanisms of PRMT oligomerization using PRMT1 as a model enzyme, which is the predominant type-I PRMT member that is responsible for the majority of arginine methylation in proteins (24). Compared with traditional structural biology methods such as X-ray crystallography, cryo-EM has the advantages of being suited for analyzing large protein complexes, having reduced radiation damage and maintaining native activity and functional state of samples and being capable of capturing multiple different structural states in one experiment (25). Herein we for the first time resolved different forms of higher-ordered oligomer structural forms of PRMT1 with cryo-EM. We performed various orthogonal biophysical and biochemical experiments to illuminate the downstream effects of oligomerization on the enzymatic activity of PRMT. Our study reveals the structural basis of PRMT oligomerization and proves that oligomerization plays an effective role in regulating the enzymatic activity of PRMTs.

## Results

### Structural elucidation of PRMT1 higher-ordered oligomers using cryo-EM

**Figure 1:**
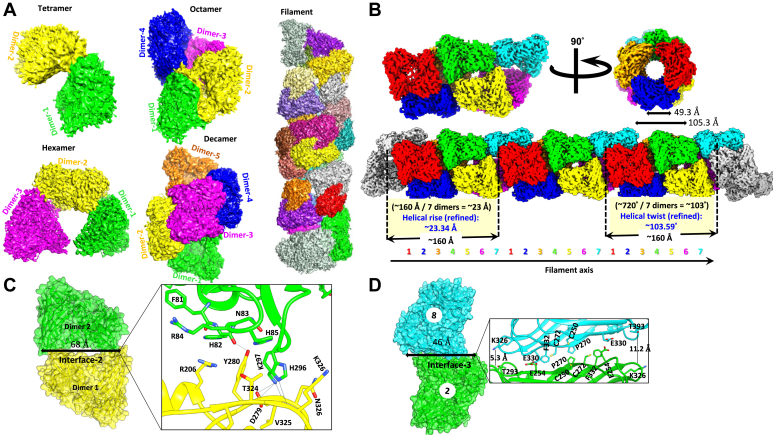
**Structural characterization of PRMT1.***A*, surface view of 3D structures of various oligomerization states of PRMT1. *B*, surface view of the PRMT1 reconstruction superimposed with the subunit models; the subunits are represented in different colors. The filament has a right-handed helical symmetry with a rise of 23.34 Å and a 103.59° twist. *C*, the PRMT1 oligomer interface 2 region depicts the interface residues and interactions. Residues located at the interface 2 are shown and labeled. The interactions are shown as *black* lines. *D*, the PRMT1 oligomer interface 3 region depicts the interface residues. Residues located at the interface 3 are shown and labeled. The interactions are shown as *black* lines. Two distances are shown, indicating that the residues are not close enough to form strong interactions.

Several lines of evidence suggest that PRMT1 can form larger molecular weight assemblies beyond the dimer form (15, 17, 20). However, the structural organization of PRMT1 oligomers and the functional consequences of the oligomerization are unknown. In this study, we solved the cryo-EM structure of the oligomeric states of PRMT1. Human PRMT1 protein was expressed in fusion with a His-MBP tag in its N-terminal. After initial purification on the Ni-NTA affinity column, the His-MBP tag was removed, followed by protein purification by SEC. The SEC profile of PRMT1 showed one broad elution peak between 0.4 to 0.5 column volume in the S200 column (Fig. S1*A*), suggesting that the majority of the PRMT1 protein is in a single oligomeric form (the elution time point is close to tetramer or hexamer) under the SEC condition. Interestingly, we did not observe the dimeric form of PRMT1 on the SEC chromatogram, as shown in the protein crystal form (13). The purified PRMT1 protein was concentrated at 0.7 mg/ml for cryo-EM structure study. The raw micrographs and 2D classification results displayed various oligomeric states of PRMT1, ranging from dimer, tetramer (dimer of dimers), hexamer (trimer of dimers), octamer (tetramer of dimers), decamer (pentamer of dimers), and even a helical filament form (Figs. 1, *A* and *B*, and S1). The 3D reconstruction revealed the cryo-EM structures of these PRMT1 forms at (near-) atomic resolution (Figs. 1, S1–S3). Impressively, some filamentous PRMT1 forms were observed at high frequency in most cryo-EM images (Fig. S1), which suggests a tendency of oligomer formation in a side-to-side manner. PRMT1 filaments has a right-handed helical symmetry with a rise of 23.34 Å and a 103.59° twist, and each helical turn consisted of ∼7 monomers with a length of ∼160 Å (Fig. 1*B*). In contrast, the hexamer previously found in the crystal structure of yeast PRMT1 (Hmt1) protein forms an enclosed hexamer (19), completely different from the side-to-side packing manner in our human PRMT1 cryo-EM structure. The previous X-ray crystal structure revealed that PRMT8 dimers assemble into helical filaments and form a stable pentamer of dimers (21). The PRMT1 filament discovered herein shows some similarity to that of PRMT8. PRMT1 filament also possesses three interfaces, referred as interfaces 1, 2, and 3 in PRMT8, for the helical filament formation. Interface 1 is involved in homodimer formation, as explained previously (13). Interface 2 is formed between each dimer-dimer and is stabilized by a set of interactions. These interactions include His296 of dimer A with a backbone of Val325 and Asp279 of dimer B. The hydrogen bond between Lys297 (dimer A) and Asp279 (dimer B) was also observed. Lastly, Tys280 (dimer A) forms a hydrogen bond with His82 (Dimer B) backbone (Fig. 1*C*). Interface 2 also comprises of Tyr81, His82, Asn83, Arg84, His85, His296, and Lys297 from one dimer and Arg206, Asn278, Asp279, Tyr280, Tyr323, Thr324, Val325, Lys326, and Leu359 from the other dimer. PRMT1 interface 2 is almost identical to PRMT8 interface 2, except for two residue changes: Lys107 (PRMT8) corresponding residue to PRMT1-Arg84 and Arg349 to the PRMT1-Lys326 (Fig. 1*D*). Dimer 1 and 4 formed interface three. Several interface residues were observed in interface 3, including Cys250, Phe332, Cys272, Glu330, Glu254, Phe332, Ala270, Tyr335, Ala293, Lys326, and Thr293; most of them are identical to PRMT8 interface three. However, unlike PRMT8 interface 3, the distances between residues in the PRMT1 interface 3 are generally not close enough to form any strong interaction.

### PRMT1 oligomerization characterized by dynamic light scattering

**Figure 2:**
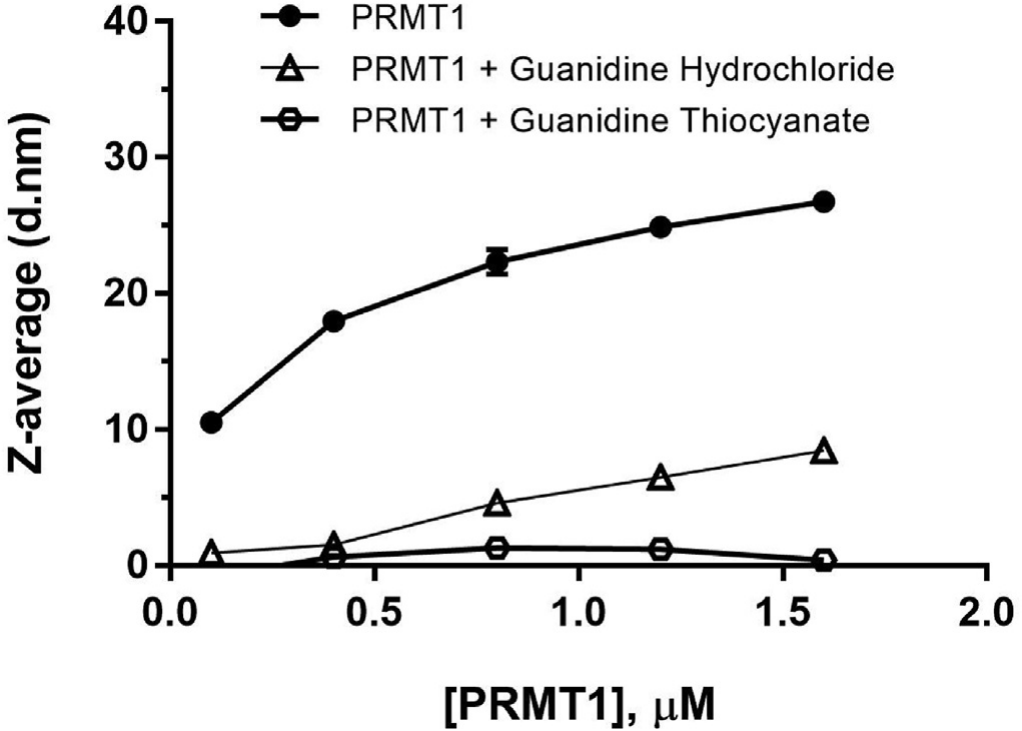
**Oligomerization of PRMT1 detection by dynamic light scattering.** Various concentrations of PRMT1 were incubated on ice for 10 min, then filtered by 0.2-micron syringe filter and collected data from the DLS. The *top* line with *black* circle indicates PRMT1 without denaturant. The *open triangle* and hexagon lines indicated PRMT1 oligomeric structure disrupted by 6 M guanidine hydrochloride and 5 M guanidine thiocyanate, respectively.

Dynamic light scattering (DLS) uses a laser to measure the particle size distribution (26). The DLS can give information regarding the heterogeneity of protein in a sample (27). In the past, DLS was used to study microbial and viral structure (28). Nowadays, DLS is a popular method for studying nanoparticles, liposomes, and exosomes (29, 30, 31, 32). Researchers have also used DLS to study the protein folding of bovine serum albumin (33). Therefore, we envisage that the DLS can be utilized to analyze the size changes of PRMT1 oligomeric compositions in an aqueous solution. We expect to see an increase in size with increasing protein concentrations. The prepared PRMT1 protein at varied concentrations was allowed to incubate in the aqueous buffer for equilibrium on ice for 10 min. The protein solution was filtered with 0.2-micron filter to remove any big interfering particles. The protein size was analyzed using a Malvern Zetasizer instrument. We took an average of three runs with about 25 scans each time to evaluate the data. When graphing the Zeta-average (Z-average) with the PRMT1 concentration, the Z-average of the protein size increased gradually with the increase in PRMT1 concentration (Fig. 2). Guanidine hydrochloride is a popular protein denaturant (34). Hence, we used it to disrupt PRMT1 oligomers. As a negative control, we incubated PRMT1 in the presence of 6 M guanidine hydrochloride to disrupt oligomer formation. As shown in Figure 2, the Z-average values of PRMT1 increased as a function of PRMT1 concentration, indicating growing oligomerization sizes. The Z-average values in the presence of guanidine hydrochloride were substantially smaller than those values without guanidine hydrochloride, attributed to the denaturing effect of the chaotropic agent. A literature reported that guanidine thiocyanate is a stronger denaturant than guanidine hydrochloride (35). Hence, we performed an additional experiment to disrupt the PRMT1 oligomers with 5 M guanidine thiocyanate. Indeed, the data showed that guanidine thiocyanate was better at denaturing PRMT1 oligomers than guanine hydrochloride. The highest Z-average obtained when disrupted with 6 M guanidine hydrochloride was 8.43 nm, but it dramatically decreased to 1.27 nm in the presence of 5 M guanidine thiocyanate (Fig. 2). The comparative data demonstrated that PRMT1 formed oligomers in the aqueous buffer, the degree of oligomerization went higher as protein concentrations increased, and the chaotropic agents guanidine hydrochloride and guanidine thiocyanate denatured PRMT1 structure and disrupted its oligomeric states.

### Mass spectrometry analysis of methylated products of PRMT1 catalysis at different oligomeric enzyme states

**Figure 3:**
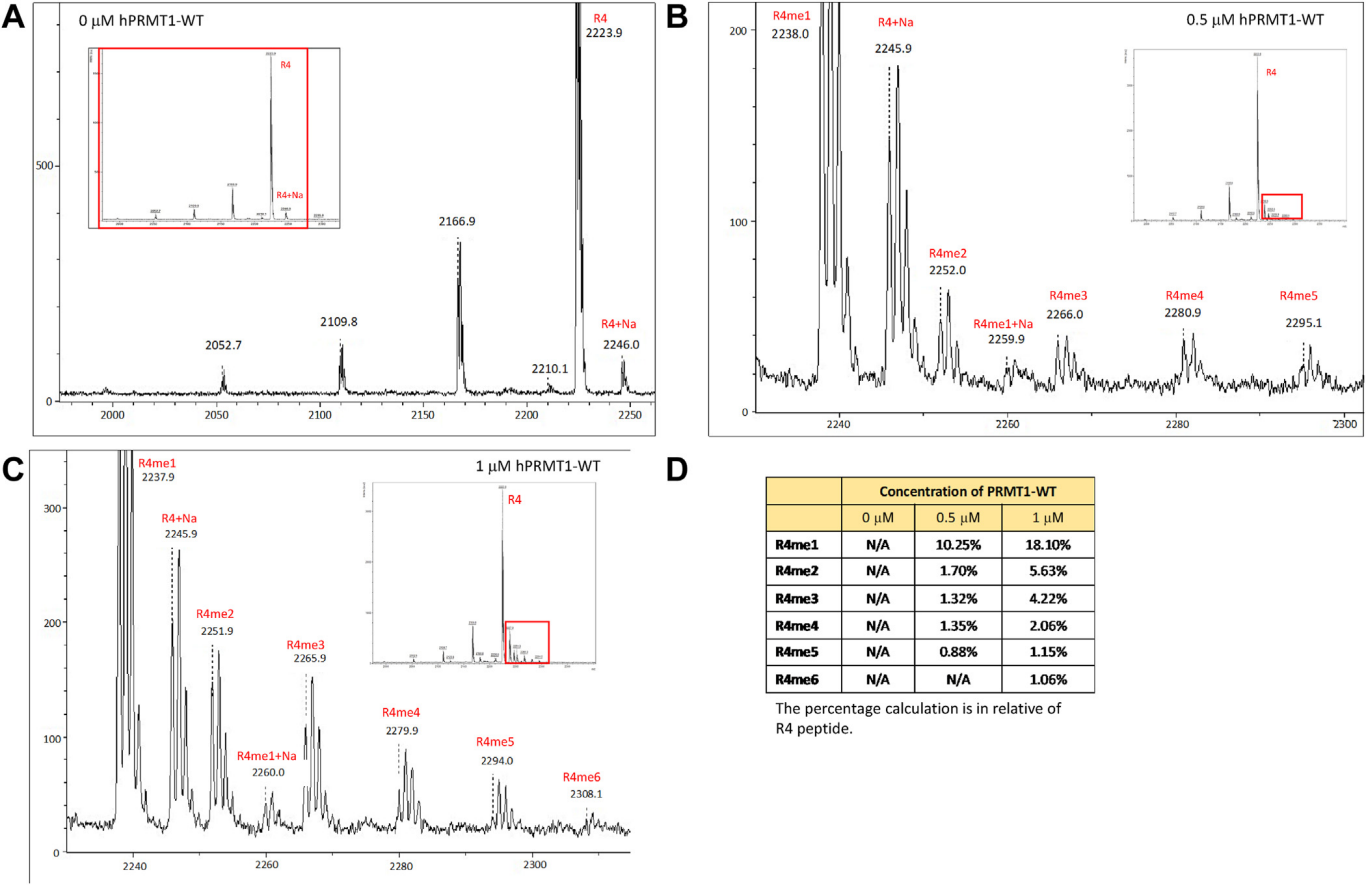
**Determine the methylation state of R4 peptide by PRMT1 high-order oligomers.** The reaction consisted of 0 μM (*A*), 0.5 μM (*B*), 1 μM (*C*) of PRMT1, 10 μM R4 peptide, and 50 μM SAM for 30 min at room temperature. The reaction was quenched with 5% TFA and subjected to MALDI analysis. *D*, the methylation percentage of individual peak in relative to R4 peptide.

**Figure 4:**
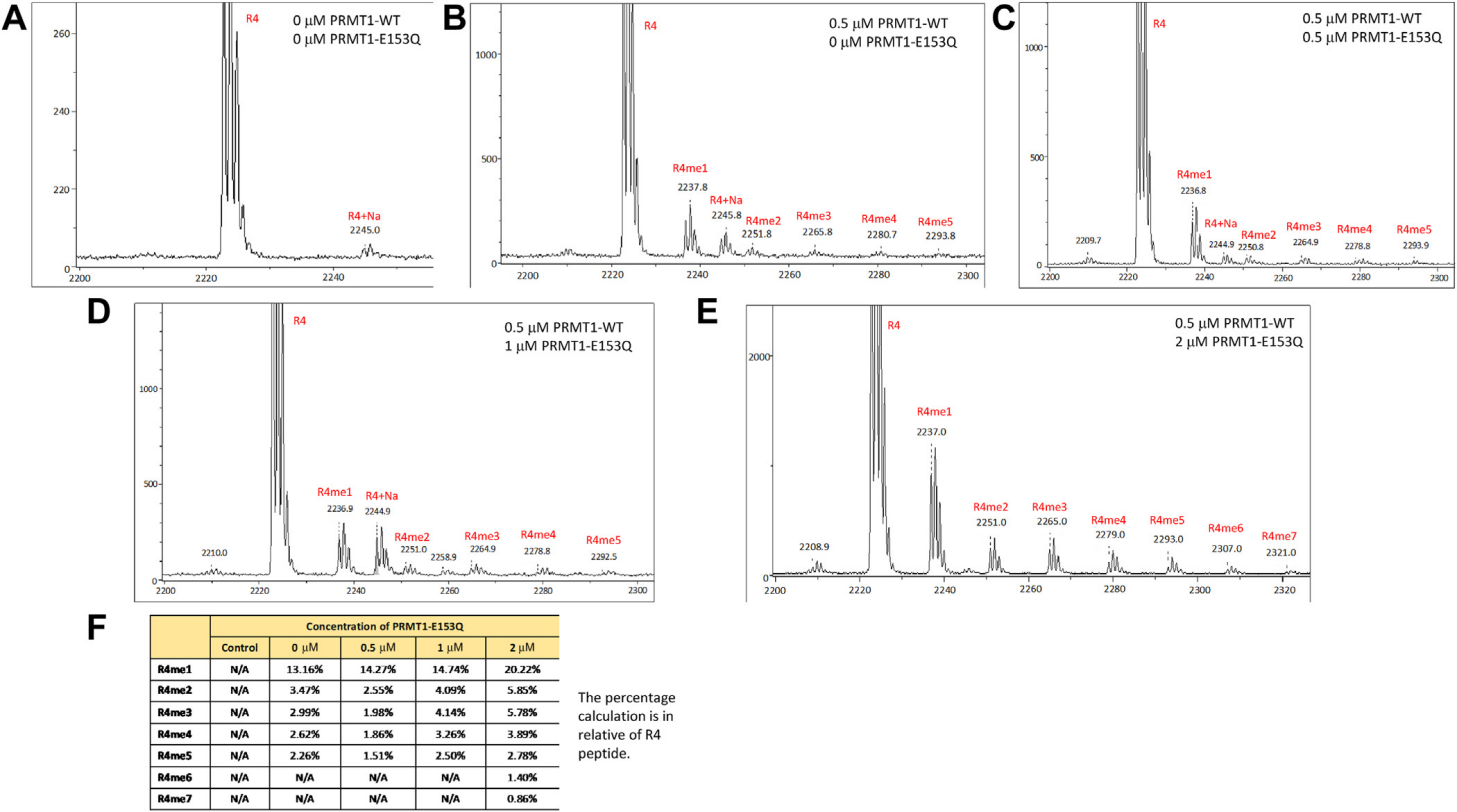
**Determine the methylation state of R4 peptide by PRMT1 and PRMT1-E153Q oligomers formation.** PRMT1 and PRMT1-E153Q was incubated on ice for 20 min before the addition of 10 μM R4 and 50 μM of SAM. The reaction was at 25 °C on water bath for 30 min before quenching with 5% TFA. MALDI analysis shown the methylation events at different concentrations of PRMT1-E153Q. *A*, no PRMT1-WT or PRMT1-E153Q was added to the reaction. *B*, 0.5 μM of PRMT1-WT and no 0 PRMT1-E153Q to test for oligomerization without PRMT1-E153Q presence. *C*–*E*, contained 0.5 μM PRMT1-WT with 0.5 μM, 1 μM, and 2 μM PRMT1-E153Q, respectively. *F*, the methylation percentage of individual peak in relative to R4 peptide.

**Figure 5:**
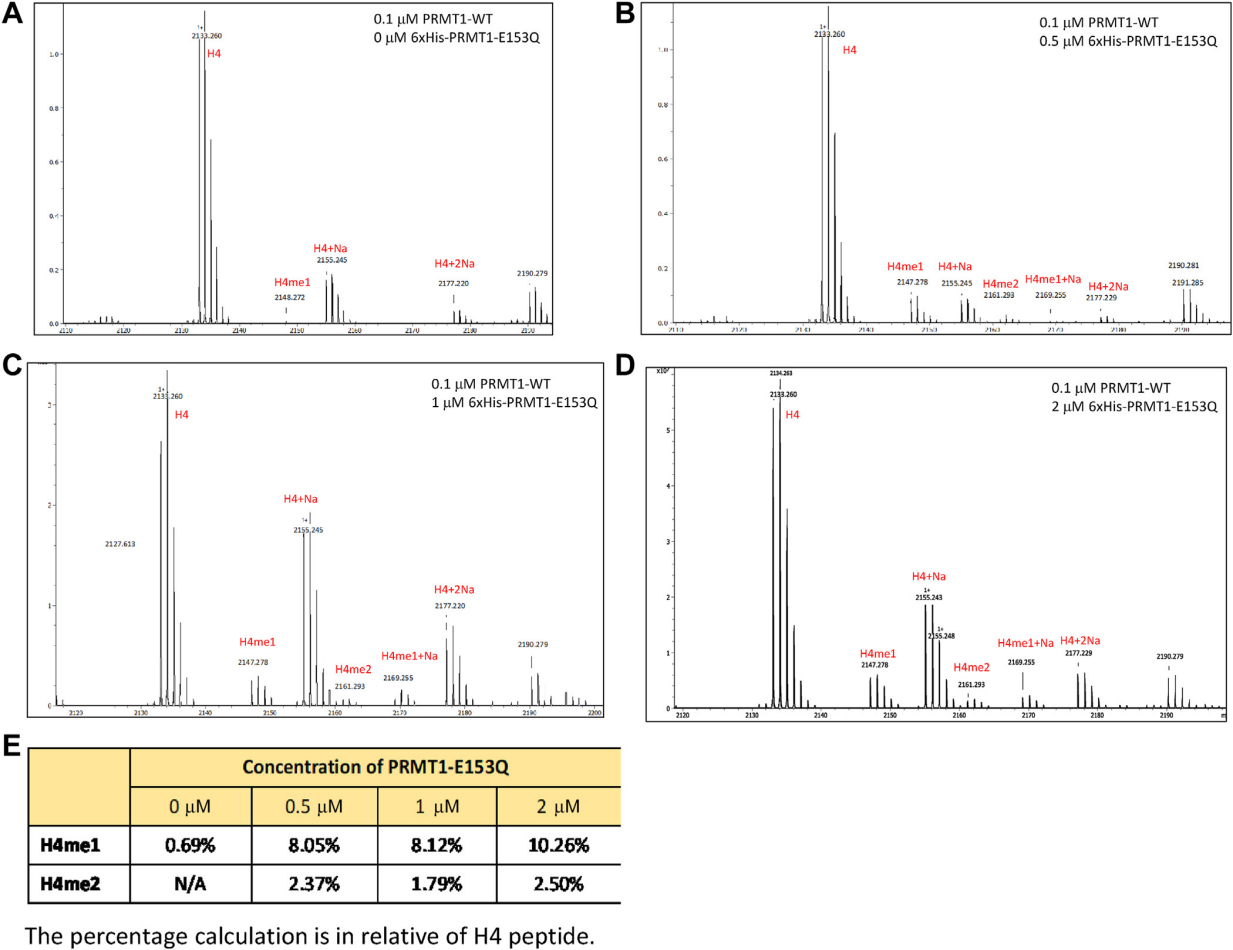
**Determine the methylation state of H4 peptide by PRMT1 and PRMT1-E153Q oligomers formation.** PRMT1 and PRMT1-E153Q was incubated on ice for 20 min before the addition of 10 μM H4 and 50 μM of SAM. The reaction was at 25 °C on water bath for 30 min before quenching with 5% TFA. MALDI analysis shown the methylation events at different concentrations of PRMT1-E153Q. *A*, 0.1 μM of PRMT1-WT and no PRMT1-E153Q to test for oligomerization without PRMT1-E153Q presence. *B*–*D*, contained 0.1 μM PRMT1-WT with 0.5 μM, 1 μM, and 2 μM PRMT1-E153Q, respectively. *E*, the methylation percentage of individual peak in relative to R4 peptide.

It is known that PRMT1 can mono- and di-methylate an arginine residue on a protein or peptide substrate sequence, which results in monomethylarginine and asymmetric dimethylarginines product states. We attempted to examine whether oligomerization promotes PRMT1 ability to generate multiple methylation states in substrates. We used a synthetic peptide with four possible arginine methylation sites for PRMT1: the R4 peptide (Ac-GG**R**GGFGG**R**GGKGG**R**GGFGG**R**GGFG). R4 peptide, SAM, and different concentrations of PRMT1 were mixed in an aqueous buffer to allow methylation to occur, and then the mixture was subjected to MALDI-MS analysis. The MALDI-MS spectra clearly demonstrated that the R4 peptide was multimethylated by PRMT1, from monomethylation to penta-methylation and hexa-methylation, even though the substrate peak was still the dominant species (Fig. 3). Comparing the activities of 0.5 μM and 1 μM PRMT1, the latter generated more numbers of multiple methylation peaks, and the hexa-methylation peak was only observed at 1 μM. These data are suggestive that oligomerization promotes the processivity of PRMT1 catalysis. In the next experiment, we examined how the addition of PRMT1-E153Q, a catalytically inactive mutant, to the PRMT1-WT solution affects the progression of the R4 peptide methylation. Our rationale is that, even though PRMT1-E153Q is catalytically dead, it can still interact with PRMT1-WT, resulting in hybrid higher oligomers. Therefore, presenting varying amounts of PRMT1-E153Q to a fixed concentration of PRMT1-WT would be another ideal way to test how oligomerization impacts on PRMT1 activity. Firstly, PRMT1-E153Q was examined for enzymatic activity and indeed, there was no methylated product detected on the MALDI-MS spectra in a PRMT1-E153Q-SAM-R4 mixture (Fig. S5*A*). In contrast, PRMT1-WT exhibited up to six methylation states in the R4 substrate (Fig. S5*B*). Next, the concentration of PRMT1-WT was kept constant at 0.5 μM, but the concentration of PRMT1-E153Q was changed (Fig. 4). Surprisingly, the amounts of all of the different methylation states increased as more and more PRMT1-E153Q protein was added. For example, comparing the reaction mixture containing 2 μM PRMT1-E153Q and the control reaction containing only PRMT1-WT, monomethylated product R4me1 increased from 13.16% to 20.22% and dimethylated R4me2 increased from 3.47% to 5.85%. Also, hexa- and hepta-methylated R4 peptides appeared when PRMT1-E153Q was increased up at 2 μM. We further tested the methylation of the H4 peptide by 0.1 μM PRMT1-WT with the presence of PRMT1-E153Q. The data displayed in Figure 5 revealed a similar trend as the R4 peptide: the methylation intensity of monomethylation on H4 peptide increased with the increase of PRMT1-E153Q concentration. In the sample without PRMT1-E153Q, second methylation on H4 peptide was not seen but was clearly observed in the presence of PRMT1-E153Q (Fig. 5*E*). This data clearly demonstrated that oligomerization plays a positive role in promoting multiple methylation of the substrate, an indicator of processivity enhancement.

### Examination of the effect of oligomerization on PRMT1 catalysis using radiometric assays

**Figure 6:**
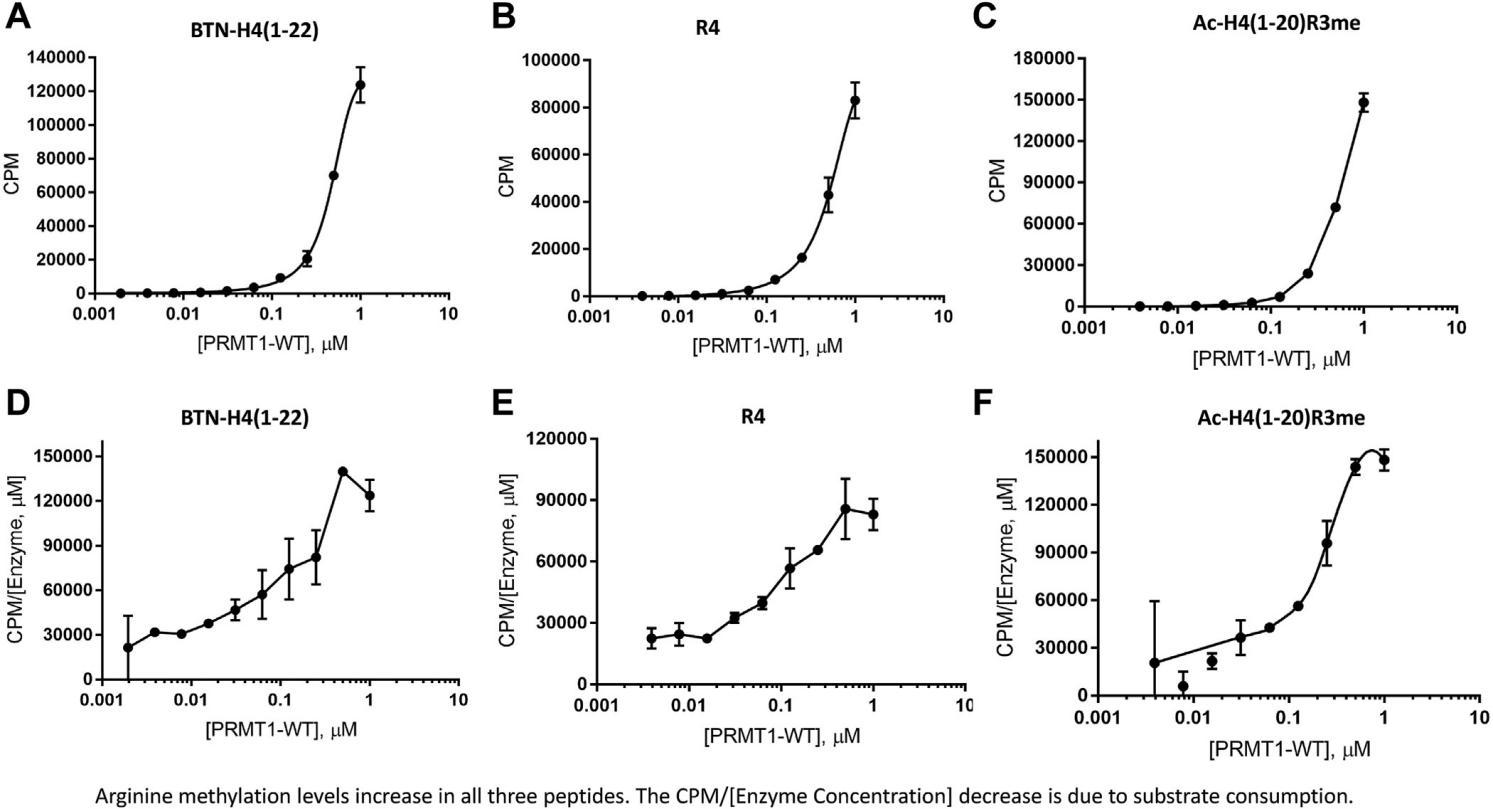
**Arginine methylation of various peptides by high-order oligomeric PRMT1-WTmeasured using the radiometric filter-binding assay**. Various concentrations of PRMT1-WT were incubated with radioactive 2 μM [3H]-SAM and 48 μM SAM and 50 μM of indicated peptides for 30 min. The reaction was quenched with 100% isopropanol. *A*–*C*, demonstrated PRMT1 methylation of peptide CPM. *D*–*F*, demonstrated CPM per monomer of PRMT1.

To further explore the effect of high-order oligomerization on PRMT1 activity, we quantified arginine methylation in BTN-H4(1–22) peptide (sequence: BTN-SG**R**GKGGKGLGKGGAKRHRKVL), R4 peptide (sequence: Ac-GG**R**GGFGG**R**GGKGG**R**GGFGG**R**GGFG), and Ac-H4(1–20)R3me (peptide sequence: Ac-SG**Rme**GKGGKGLGKGGAKRHRK) peptides. We used the phosphocellulose P81 filter paper–binding assay to measure the catalytic activity of PRMT1 at different concentrations on individual peptides. Each reaction mixture was composed of PRMT1-WT at varied concentrations, at fixed concentrations of SAM and peptide. The reaction mixture was quenched with isopropanol, transferred to the P81 filter paper, and washed with sodium bicarbonate buffer (pH 9). The filter paper was placed in a scintillation vial, and after air drying, an Ultima Gold scintillation cocktail was added for scintillation counting. The measured count-per-minute (CPM) values correlated directly with the amounts of methylated peptide products. In a typical enzymatic catalysis, the product should be formed linearly as a function of enzyme concentration. However, the methylated product formation in PRMT1 catalysis did not follow a linear trend as a function of PRMT1 concentration (Fig. 6, *A*–*C*). Instead, the activity-concentration plotting displayed an exponential-like curve for all the three tested peptides. The catalytic turnover rate (*i.e.*, *k*_cat_) of PRMT1 catalysis was calculated as the slope of data curves of Figure 6, *A*–*C* (*i.e.*, the CPM values of the methylated peptides divided by PRMT1 concentrations) and displayed in Figure 6, *D*–*F*. For all of the three peptides, the *k*_cat_ values were not constant; they all went up as the enzyme concentrations increased. The data further substantiated that oligomerization, as a result of increased enzyme concentrations, upregulates PRMT1 activity. These results coincide well with a preliminary study we reported before (20). It was notable that the catalytic turnover rate started to level off or even slightly decrease when PRMT1 concentrations were greater than ∼1 μM (Fig. 6, *D*–*F*). The slowdown in *k*_cat_ increase was most likely because SAM or the peptide substrate were overly consumed at high enzyme concentrations.

**Figure 7:**
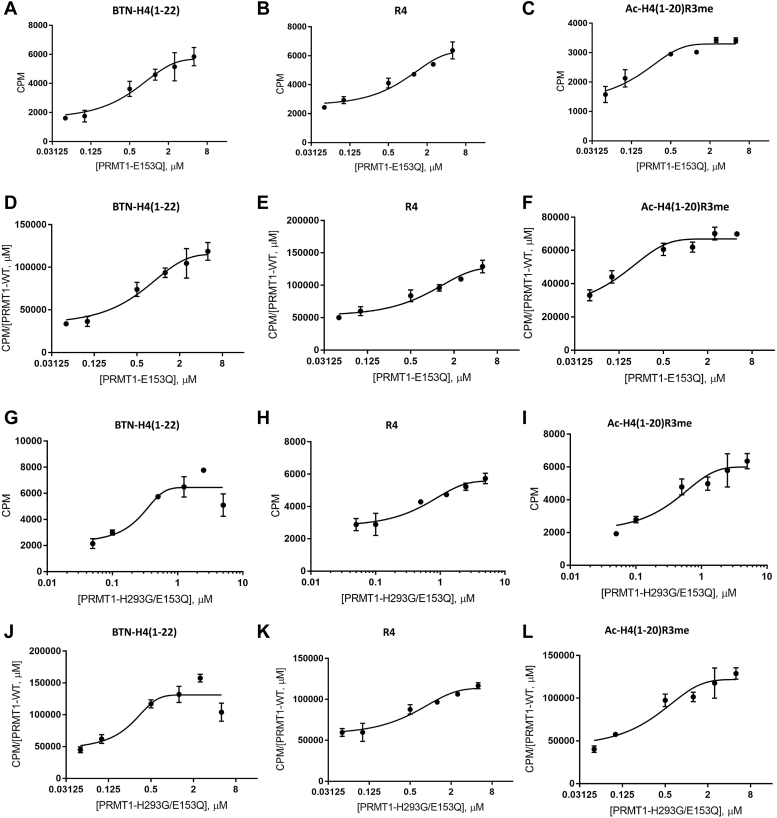
**Arginine methylation of various peptides by high-order oligomeric PRMT1-WT and PRMT1 mutants.** Filter-binding assay was used to characterize the methyltransferase activity. Firstly, 0.05 μM of PRMT1 was incubated with various concentrations of PRMT1-E153Q or PRMT1-H293G/E153Q on ice for 20 min. Afterward, 2 μM radioactive [3H]-SAM and 48 μM SAM and 50 μM of indicated peptides was added and incubated for 30 min before quenching the reaction with 100% isopropanol. *A–C*, showed PRMT1 methylation of peptides in CPM in the presence of PRMT1-E153Q. *D–F,* demonstrated CPM per monomer of PRMT1 in the presence of PRMT1-E153Q. *G–I*, showed PRMT1 methylation of peptide in CPM in the presence of PRMT1-H293G/E153Q. *J–L*, demonstrated CPM per monomer of PRMT1 in the presence of PRMT1-H293G/E153Q.

Next, we added the catalytically inactive mutant PRMT1-E153Q to a PRMT1-WT reaction mixture to further understand how oligomerization affects the catalytic activity of PRMT1. Constant PRMT1-WT concentration and various PRMT1-E153Q concentrations were maintained in the aqueous buffer prior to adding peptide and SAM to initiate the reaction. The reaction was quenched with isopropanol. The reaction mixture was loaded on the P81 filter paper that was air dried, washed, and immersed in scintillation cocktail for scintillation counting. Since the catalytically inactive PRMT1-E153Q mutant had only very weak activity, slightly above the background level (Fig. S6, *A* and *B*), any observed methylation activity would be attributed to PRMT1-WT, and any of PRMT1-E153Q induced effects would be due to its oligomerization with PRMT1-WT to form higher-order oligomers. Consistent with the MALDI-MS characterization, the methylation levels of all the tested peptides increased as more PRMT1-E153Q proteins were added to PRMT1-WT solution (Fig. 7, *A*–*C*). Correspondingly, the *k*_cat_ of PRMT1-WT, represented by the CPM numbers divided by PRMT1-WT concentration, also increased as a function of PRMT1-E153Q concentration (Fig. 7, *D*–*F*). We were slightly concerned that, even though very weak, PRMT1-E153Q still exhibited a tiny amount of activity (Fig. S6, *A* and *B*) which might complicate the data interpretation. To eliminate this possibility, we created a double mutant (PRMT1-H293G/E153Q) to further abolish PRMT1 activity. The data showed that the activity of PRMT1-H293G/E153Q was even weaker than PRMT1-E153Q, greatly diminished to near background level (Fig. S6, *C*–*E*). We then tested PRMT1-WT activity in the presence of various concentrations of PRMT1-H293G/E153Q (Fig. 7, *G*–*L*). The methylated product amount in the CPM value gradually went up as the concentrations of the double mutant increased. Furthermore, the *k*_cat_ of PRMT1-WT, represented by the CPM numbers divided by PRMT1-WT concentration, also increased as a function of PRMT1-H293G/E153Q concentration. These increasing trends were the same as that of PRMT1-E153Q. Altogether, these data supported that oligomerization enhances PRMT1 activity. Again, similar to Figure 6, *D*–*F*, the increase in *k*_cat_ started to slow down a bit at >1 μM concentrations of PRMT1-E153Q or PRMT1-H293G/E153Q, which is likely due to substrate overconsumption under these conditions.

**Figure 8:**
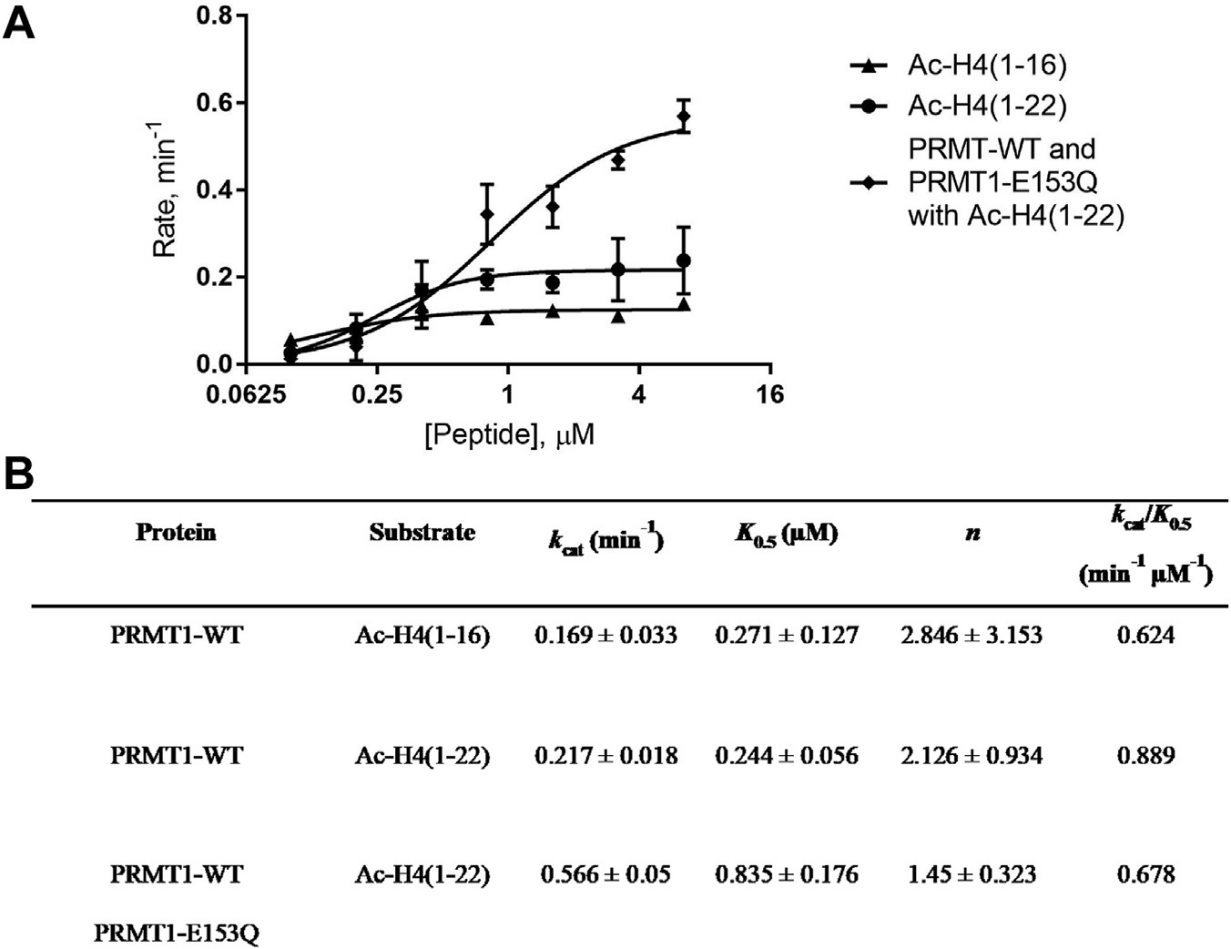
**Steady state kinetic characterization of PRMT1 catalysis in oligomeric states.** Radioactive kinetic parameters were measured using filter-binding assay. The reaction mixture consisted of 0.05 μM PRMT1-WT, 15 μM ^14^C-SAM, and various concentrations of Ac-H4(1–16) or Ac-H4(1–22) peptide. The reaction time was 15 min at 30 °C and quenched with 100% isopropanol. For the reaction with PRMT1-E153Q, 0.05 μM PRMT1-E153Q was incubated on ice with PRMT1 for 20 min equilibration before adding SAM and peptide. *A*, kinetic parameters of oligomeric states in graphical form. *B*, representation of the kinetic parameters in *k*_cat_, *K*_0.5_, and *k*_cat_/*K*_0.5_.

To gain further insights into the effect of oligomerization on the PRMT1 activity, we measured the steady-state kinetic parameters of different peptide substrates under PRMT1 catalysis using the radiometric assay. We chose two histone H4 peptides as substrate: Ac-H4(1–16) (peptide sequence: Ac-SG**R**GKGGKGLGKGGAK) and Ac-H4(1–22) (peptide sequence: Ac-SG**R**GKGGKGLGKGGAKRHRKVL). The sequence length of Ac-H4(1–16) is shorter than the Ac-H4(1–22), so likely the former peptide binds to PRMT1 weaker and gets more easily released into the bulk solution than the latter. A constant concentration of PRMT1-WT was mixed with various concentrations of peptide and a constant concentration of radioactive SAM. After quenching the reaction, the reaction mixture was loaded onto the P81 filter paper that was washed, air-dried, and soaked in scintillation cocktail for scintillation counting. The rates of methylation as a function of the peptide concentration were plotted in Figure 8*A* and fit with the Hill equation to obtain catalytic parameters *k*_cat_ and *K*_0.5_ (Fig. 8*B*). The *k*_cat_ of PRMT1 for Ac-H4(1–16) peptide is 1.3-fold lower than Ac-H4(1–22) (Fig. 8, *A* and *B*), and the *K*_0.5_ of Ac-H4(1–16) is greater than Ac-H4(1–22). The *k*_cat_/*K*_0.5_ of Ac-H4(1–22) was 1.4-fold higher than Ac-H4(1–16) peptide. These comparative data demonstrated that PRMT1 was more efficient for methylating the longer peptide Ac-H4(1–22) than Ac-H4(1–16), a conclusion consistent with that reported previously by the Thompson lab (36). The changes in *k*_cat_ and *K*_0.5_ values suggest that the greater activity in the longer Ac-H4(1–22) peptide was mechanistically contributed by both substrate binding and catalytic turnover enhancement.

We next examined how the presence of the catalytically inactive PRMT1-E153Q mutant affects steady-state kinetics of PRMT1-WT catalysis. PRMT1-E153Q and PRMT1-WT were incubated in the aqueous buffer on ice before adding various concentrations of Ac-H4(1–22) peptide and a constant concentration of [^3^H]-SAM. The reaction and the workup procedure were the same as mentioned above. The initial velocity is presented in Figure 8*A*, and the calculated kinetic parameters are shown in Figure 8*B*. The *k*_cat_ of the PRMT1-WT/PRMT1-E153Q hybrid complex in methylating Ac-H4(1–22) peptide was 2.6-fold higher than just PRMT1-WT alone, confirming the strong positive regulatory effect of oligomerization on catalysis. Interestingly, the *K*_0.5_ value of the PRMT1-WT/PRMT1-E153Q mixture was 3.4-fold higher than PRMT1-WT alone, which can be explained by the fact that adding PRMT1-E153Q to the reaction mixture of PRMT1-WT poses more competing binding sites for the substrate, and so higher amounts of substrates are needed to saturate the active site of PRMT1-WT enzyme.

### Effect of PRMT1 oligomerization on substrate release

**Figure 9:**
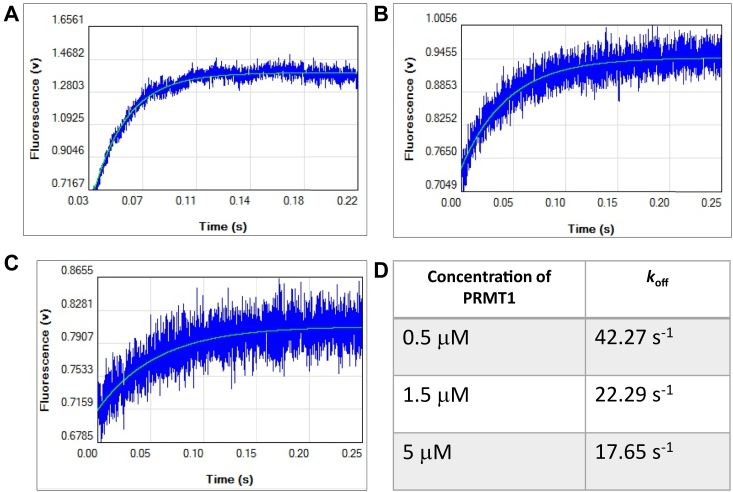
**Oligomerization helps PRMT1 retain its substrate.** The experiment was carried out by the SX20 stopped-flow spectrometers with (*A*) 0.5 μM, (*B*) 1.5 μM, and (*C*) 5 μM of PRMT1. The PRMT1 was premixed with 0.8 μM H4FL peptide. The mixture was diluted with the reaction buffer for a 1:25 dilution when the reaction was run. *D*, data summary of *k*_off_.

We next utilized the stopped-flow technique to measure the dissociation rates of peptide substrate from PRMT1 at different PRMT1 concentrations. We used a rapid dilution experiment with stopped flow fluorescence to measure the dissociation rate of H4 peptide from PRMT1. A fluorescently labeled H4 peptide, H4FL (Ac-SG**R**GKGGKGDpr(FL)GKGGAKRHRK. FL stands for fluorescein), was previously shown to exhibit fluorescence intensity change upon PRMT1 association and dissociation (20, 37, 38). We reasoned that the changes of fluorescence intensity can be used to measure dissociation rate of H4 substrate. In the experiment, the H4FL was premixed with PRMT1 and loaded into the sample chamber of the stopped flow equipment. The mixture was rapidly mixed with an access buffer which initiated the dissociation of H4FL from PRMT1 on the millisecond scale. As shown in Figure 9, , *A*–*C*, the fluorescence of H4FL increased as the time progressed to the release of H4FL to the bulk solution. The averaged data curves were fitted to the single exponential equation to yield dissociation rate constant *k*_off_. The *k*_off_ was 42.27 s^−1^ at 0.5 μM of PRMT1, the *k*_off_ was 22.29 s^−1^at 1.5 μM of PRMT1, and the *k*_off_ was 17.65 s^−1^ at 5 μM of PRMT1 (Fig. 9*D*). Clearly, the *k*_off_ decreased as PRMT1 concentration increased. This data concluded that oligomerization increased the residence time of peptide substrate in the PRMT1 protein.

### Peptide-protein docking reveals the binding modes of H4(21)-peptide to PRMT1 oligomers

**Figure 10:**
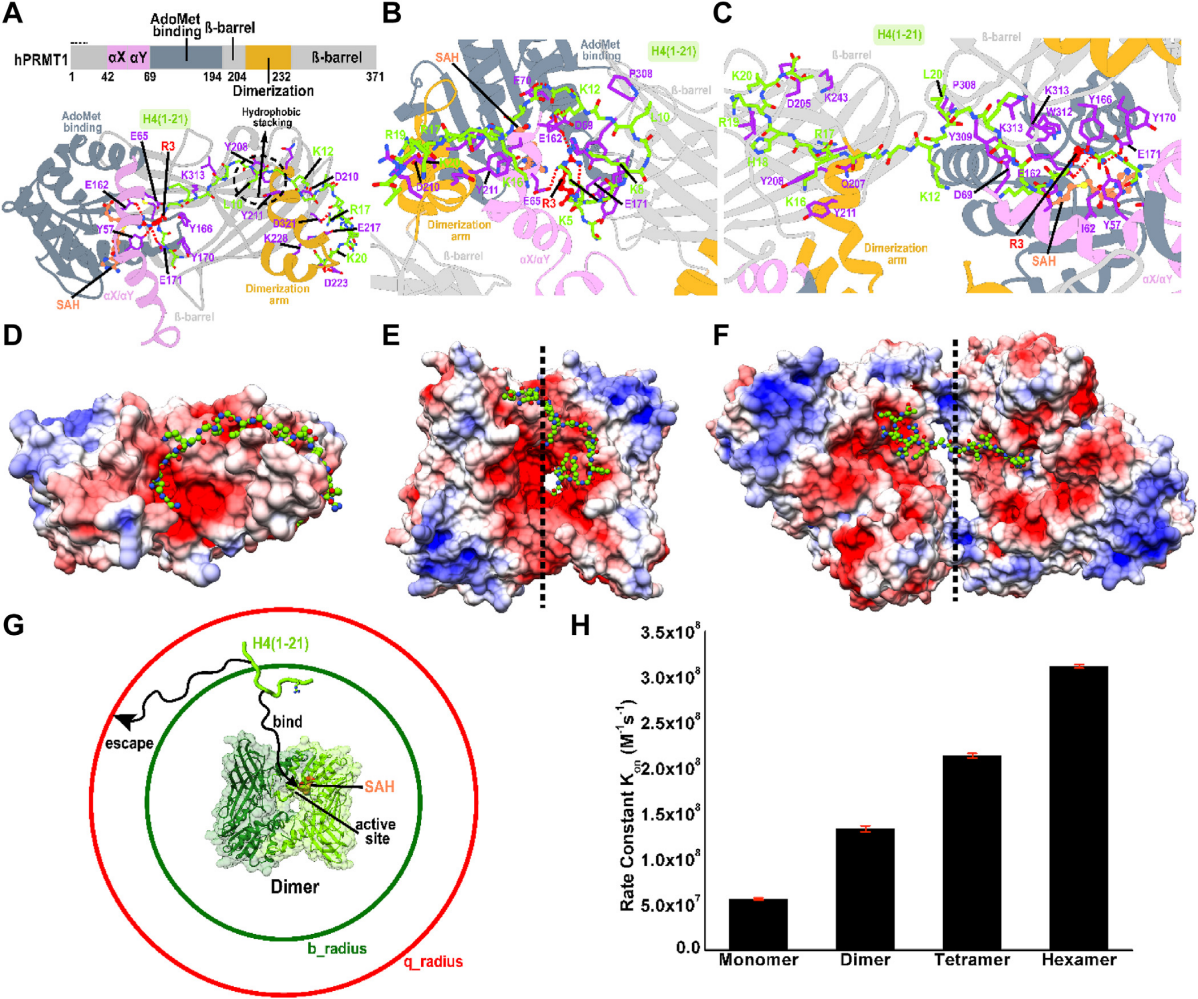
**Binding modes and kinetics of H4(21)-peptide association to PRMT1 oligomers.** Zoomed-in view of the binding contacts formed by H4(21) peptide in its primary docking configuration with (*A*) monomer, (*B*) dimer, and (*C*) tetramer of PRMT1. The oligomers in the *top* panel are colored by domains as indicated in the inset of panel a. H4(21) peptide is depicted in *light green*. Docking conformations of the H4 peptide and surface electrostatics of the PRMT1 complexes for (*D*) monomer, (*E*) dimer, and (*F*) tetramer. The electrostatic potentials were computed with APBS and mapped onto the molecular surfaces in the range from -5k_b_T/e (*red*) to +5k_b_T/e (*blue*). *G*, a schematic representation of the BrownDye algorithm with decision boundaries being marked in *red* and *dark green*. *H*, Second-order rate constants *k*_*on*_ for H4 peptide binding to the different oligomers of PRMT1 as estimated from Brownian dynamics calculations.

To understand the detailed binding interactions of the H4(21)-peptide with human PRMT1 monomer, dimer, and tetramer, we employed peptide–protein docking with the Rosetta modeling package. Specifically, we used the Flex-Pep-Dock *ab initio* protocol in Rosetta to compute 100,000 binding poses (decoys) for each of the three complexes. Distance constraints were applied to ensure the PRMT1 active site residues E162 and E171 remained bound to R3 of the H4-peptide. Thus, all generated conformers were stably bound to one enzymatic active site. The decoys were subsequently filtered based on their Rosetta scores and clustered by RMSD to the initial model, which was based on the X-ray structure of PRMT1 monomer (PDB ID: 1OR8) (4). The lowest free energy conformer from the most populated cluster was selected to represent the structure of the H4(21)–PRMT1 monomer, dimer, and tetramer complexes, respectively. Importantly, the docking results revealed that H4 adopts distinct binding conformations in the different PRMT1 oligomeric states (Fig. 10, *A*–*C*).

Upon exiting the active site of the PRMT1 monomer, the flexible H4 peptide chain is directed along the surface of the β-barrel domain toward the PRMT1 dimerization arm. The H4 extended tail region wraps around the dimerization domain. Two aromatic residues from the dimerization arm, Y208 and Y211, bind the L10 residue from H4 and are key for the observed binding mode. Additional interactions contributing to the stable H4-peptide binding include salt bridges (*e.g.*, K12-D210, R17-D321), polar residue hydrogen bonding (K16-N218, A15-D210), and hydrophobic contacts (V21-C226) along the entire back surface of the dimerization domain (Fig. 10*A*). These findings underscore the essential role of the dimerization domain, not only for PRMT oligomerization but also for accommodating the extended tail region of the H4 substrate. Predictably, the catalytic activity of the PRMT1 monomer decreases when the dimerization domain is absent. By contrast, in the PTMT1 dimer, the H4-peptide cannot access the back surface of the dimerization arm. Instead, the tail region of the H4 substrate is redirected along the interface of the two monomers. The resulting conformation is stabilized by residue contacts with the front of the dimerization arm, the αX/αY, and cofactor binding domains of the opposing PRMT1 monomer (Fig. 10*B*). In this orientation, L10 makes hydrophobic contacts with P308. Yet, the Y208 and Y211 residues still play a critical role in anchoring H4 to the dimerization arm.

The H4 binding mode with the PRMT tetramer is distinct from both the monomer and the dimer complexes. Upon exiting the active site, the H4 peptide chain extends across the gap between the two dimers, reaching toward the β-barrel domain of one of the opposing monomers and completely bypassing the dimerization arm. Bridging residues (13–15) are glycine and alanine, while the four tail H4 residues (16 through 20) play a pivotal role for anchoring the substrate to the opposing dimer primarily through salt bridge interactions (Fig. 10*C*). Thus, the tetramer binding mode rationalizes the weaker PRMT1 binding observed in the radiometric assays for Ac-H4(1–16) relative to Ac-H4(1–21). Moreover, stabilization of the H4 tail region in higher-order oligomers stems from PRMT1 structural elements that are distal from R3 and the active site E162 and E171. Consequently, a mutant PRMT1 monomer lacking the direct R3 binding to the active site could still engage in cooperative binding through the H4 tail region. This could explain the increased binding affinity of the H4 peptide to PRMT1-WT in the presence of the PRMT1-E153Q variant (E162Q in the human sequence).

Another key observation from our computational models is that H4–PRMT1 association is driven primarily by favorable electrostatic interactions. Besides glycine residues imparting flexibility to the H4 chain, there are positively charged residues forming favorable salt-bridge interactions with numerous negative residues on the PRMT surface. To address the electrostatic contribution to substrate binding, we used the Adaptive Poisson-Boltzmann Solver (APBS) and computed electrostatic potentials for the PRMT1 monomer, dimer, tetramer, and hexamer, respectively. The electrostatic potentials were mapped onto the molecular surface of each PRMT1 oligomer (Fig. S7). We show that the H4 peptide is splayed on the oligomer surfaces, exclusively following patches of negative electrostatics. Moreover, the size of the negative electrostatics cavity increases from monomer to dimer, tetramer, and hexamer. Our docking results (Fig. 10, *A*–*C*) show that across all PRMT1 oligomers, the H4 peptide consistently aligns with the regions of negative electrostatics. Thus, electrostatic forces play a pivotal role in determining the most favorable binding orientations. In addition to favorable binding, increases in the overall negative electrostatic potential in the higher order PRMT oligomers significantly contribute to electrostatic substrate steering into the enzymatic active sites, directly impacting the catalytic rates.

To quantify the effect of electrostatic steering on the H4-peptide binding kinetics, we computed *k*_*on*_ rates for the formation of the monomer, dimer, tetramer, and hexamer complexes using Brownian dynamics simulations. The simulations were conducted with the Browndye code and modeled the diffusive encounters between the H4 and the PRMT1 oligomers and estimated the binding rates based on defined distance criteria for binding (<b_radius) and escape (>q_radius) as shown schematically in Figure 10*G*. To achieve sufficient statistical sampling, 250,000 Brownian dynamics trajectories were computed per simulation system. The calculated *k*_*on*_ values for PRMT1 monomers, dimers, tetramers, and hexamers are 5.65 × 10^7^ M^−1^s^−1^, 1.33 × 10^8^ M^−1^s^−1^, 2.14 × 10^8^ M^−1^s^−1^, and 3.12 × 10^8^ M^−1^s^−1^, respectively (Fig. 10*H*). The most significant increase in *k*_*on*_ is observed from PRMT1 monomer to dimer, as the additional PRMT1 monomer unit provided regions of negative electrostatics accessible to the H4 peptide tail. Thus, electrostatic steering is key for the observed rate enhancement upon PRMT multimerization. However, this cooperative effect becomes less pronounced as we go to the tetramer and hexamer due to the limited length of the H4 peptide. Nevertheless, in line with radiometric assay measurements, these calculations demonstrated a consistent increase in *k*_*on*_ rates with higher degrees of oligomerization. Thus, we infer that the observed rate enhancement stems from the enhanced electrostatic interactions in the dimer, tetramer, and hexamer species.

We examined the impact of mutations on the H4(1–21) peptide binding to different PRMT1 oligomers. The derived Rosetta ddG scores serve as an indicator of the mutation's impact on the peptide binding free energy. Increased positive values indicate a significant loss of peptide binding after mutation, whereas minimal values exert a reduced influence. The mutations in the active site and dimerization arms of PRMT1 structures exhibited larger ddG scores (Fig. S8 and Table S2). The mutation with large positive value can disrupt the peptide binding with PRMT1 oligomers. Further, experimental analysis of these mutational studies may be substantial to identify the loss of peptide binding to PRMT1 oligomers.

## Discussion

PRMT1 is the predominant member of the human PRMT family. Crystallographic studies reveal PRMT1 forms a stable homodimer, which is the basic unit of the active enzyme, and the disruption of homodimer formation results in the inactive enzyme (13). Beyond dimerization, several previous studies support the idea that PRMTs, including PRMT1, are capable of forming higher-order oligomeric forms (13, 14, 15, 16, 17, 19, 20, 21, 22, 23, 39, 40). The structural mechanism and functional consequences of PRMT higher-ordered oligomerization are poorly defined. Our current cryo-EM imaging for the first time comprehensively captured the various structural forms of the higher-order oligomers of PRMT1, which includes tetramer (dimer of dimers), hexamer (trimer of dimers), octamer (tetramer of dimers), decamer (pentamer of dimers); and impressively, the oligomerization can extend PRMT1 into a helical filament. Observation of multiple oligomeric states of PRMT1 suggests that the higher-order oligomerization is highly dynamic. Of comparison, on the elution profile of SEC analysis of PRMT1, only a single major peak was observed at an elution time point approximately matching either the tetrameric or hexameric form (Fig. S1*A*). One explanation for the difference in oligomeric observations between cryo-EM and SEC experiments is that the various oligomeric forms of PRMT1 in the aqueous solution are faithfully preserved during the vitrification process and captured later in the EM imaging stage, while the SEC experiment involves a harsher condition (*e.g.*, potential interference by high column pressure, mobile phase turbulence, interaction with stationary phase matrix) and thus many meta-stable oligomeric forms of PRMT1 may have been disrupted during the elution. Indeed, our other unpublished SEC data also suggest that higher orders of oligomerization can occur at high PRMT1 concentrations (data not shown). The oligomeric states of PRMT1 likely are influenced by its residing contexts and experimental conditions, such as temperature, protein concentration, pH, and ionic strength, *etc.* (21). The ability to dynamically change the oligomerization state may provide a functional regulation of PRMT activity in the cell. The spatial arrangement of the higher-ordered human PRMT1 is different from the previously reported yeast PRMT1 structure (19). Interestingly, the filamentous structure of human PRMT1 is similar to that of PRMT8 observed using X-ray crystallography (21). Comparing PRMT1 and PRMT8 filaments, interface 2 proposed by the PRMT8 filament structure is critical and conserved. Asp276, His296, and Lys297 in interface 2 were not reported previously but played important roles in interface two. Asp279, His296, and Lys297 are evolutionarily highly conserved across the species, suggesting the importance of these residues in the formation of PRMT1 oligomers (Fig. S4).

Our previous study using chemical crosslinking demonstrates that an increase in the concentration of PRMT1 enzymes leads to the formation of more oligomeric forms (20). In this study, we utilized DLS as a physically mild method to examine the oligomer changes, and it was clearly proved that PRMT1 forms higher-order oligomers in a concentration-dependent manner. We further showed that the oligomer formation can be disrupted by guanidine denaturing agent. The enzymatic activity of PRMT1 was seen to increase as higher-order oligomeric structures were formed. This behavior holds true in all tested peptides, including BTN-H4(1–22), R4, and Ac-H4(1–20)R3me. The conclusion that the increase in the oligomer formation of PRMT1 increases its catalytic activity is solidly supported by the experimental data of increased *k*_cat_ at higher PRMT1 concentrations and also by the fact that even the addition of the inactive PRMT1-E153Q mutant was able to enhance the methyltransferase activity of PRMT1-WT through oligomerization. As regards the oligomer effect of PRMT1 by adding PRMT1-E153Q to the reaction, the *k*_cat_ of PRMT1-WT/PRMT1-E153Q mixtures was 2.6-fold higher than the PRMT1 alone. This data was consistent with a previous literature report (41). Comparing the steady-state kinetic parameters of both Ac-H4(1–16) and Ac-H4(1–22) in PRMT1 catalysis, Ac-H4(1–22) showed a greater *k*_cat_ and lower *K*_0.5_ (equivalent to *K*_m_ in Michaelis-Menten kinetics). This data supports that the C-terminal residues of the H4 peptide contribute to the PRMT1 substrate binding and catalysis, which corroborates the previous study by Osborne *et al.* (36). The key binding role of the C-terminal residues of the H4 peptide in PRMT1 interaction was also clearly seen in our modeling (Fig. 10*C*). The greater *k*_cat_ Ac-H4(1–22) than Ac-H4(1–16) also suggests that the C-terminal residues of the substrate may allosterically regulate the transition state of the active site of PRMT1, in addition to enhancing the binding interaction.

Analysis of the products of peptide substrate methylation with MALDI-MS at different concentrations of PRMT1 demonstrated that the methylation states of the substrates were greatly affected by PRMT1 oligomerization. The higher order of oligomerization of PRMT1 (promoted by increasing PRMT1-WT enzyme concentrations or by the addition of PRMT1-E153Q mutant to the WT enzyme), the higher the methylation states were seen on the substrate peptides. In our experimental condition, the majority of the R4 peptides were in the unmethylated form, and only about 10% of the peptides are mono- and multi-methylated (Figs. 3 and 4). Importantly, di- and multi-methylated products were clearly seen, which hints the monomethylated intermediate is preferably further methylated than the unmethylated substrate. This trend is also observed with the H4 peptide, where monomethylating event occurred at least 4-fold higher than dimethylation (Fig. 5). These data suggest that oligomerization enhances the processivity of PRMT1 in substrate methylation. Our previous transient kinetics studies show that the active site of PRMT1 follows a distributive model of catalysis for mono- and di-methylation, which is dictated by the ordered cofactor and substrate binding and release kinetics in the catalysis (42). Putting the two factors together, we propose a catalytic scheme that the substrate arginine has to be evacuated from the active site of PRMT1 for SAH-SAM exchange before next round of methylation reaction, that is, a distributive active-site catalysis. Meanwhile, the oligomerization renders PRMT1 with the ability to perform certain degrees of processive methylation: some of the monomethylated intermediate substrate, after leaving the active site of one protomer, are bound to proximal active sites within the PRMT1 oligomer complex, without being fully dispersed into the bulk solution. Such a proposal would match well with the partially processive methylation behavior observed previously by Thomson et al. (43).

The increased *k*_cat_ of PRMT1 at higher oligomeric states implies that oligomerization may allosterically facilitate the transition-state formation in the active site. As mentioned above, the C-terminal residues of the substrate may also allosterically regulate the transition state of the active site of PRMT1. These allosteric factors may explain why the steady-state kinetics data of PRMT1 catalysis fit well only to the Hill equation, but not the classical Michaelis–Menten model (44, 45, 46). The dissociation rates of H4FL peptide from PRMT1 in the stopped-flow fluorescence measurement were smaller at higher oligomeric states (Fig. 9), demonstrating that the H4 substrate is retained longer in the high-order oligomeric states of PRMT1. It is foreseeable that oligomerization enables PRMT1 to better retain its substrate on the enzyme’s surface for the subsequent methylation event following the initial mono-methylation event. Furthermore, our molecular modeling shows that the association rate constants of H4(1–21) peptide binding to PRMT1 increase at higher oligomeric states due to enhanced favorable electrostatics in the higher-order oligomers. (Fig. 10*H*). Thus, adjacent protomers in oligomeric PRMT1 help immobilize the substrate on the enzyme surface, which would increase local concentrations of a substrate on the enzyme surface, leading to enhanced enzymatic activity. Taken together, oligomerization enhances substrate binding and also allosterically improves the transition-state formation, which synergistically contributes to the enhancing effect of oligomerization on PRMT enzymatic activity.

In conclusion, our structural study and combined biophysical and biochemical data demonstrated that PRMT1 exists in different oligomeric states in the aqueous solution, and PRMT1 becomes more active when it is at a high concentration due to higher-order oligomer formation. PRMT1 mutants, even those that lose activity, can form oligomers with WT PRMT1 and thereby enhances its activity. Our study establishes higher-ordered oligomerization as a molecular mechanism for PRMT activity regulation. PRMT oligomerization and the functional consequence we herein found pose a confounding implication for PRMT activity regulation in physiology and pathology. PRMT mutation has been found in a number of tumors (https://portal.gdc.cancer.gov/). Especially, some studies have shown that cellular PRMT1 level can be present at submicromolar concentrations (22, 47). In the scenario of heterologous mutations, some PRMT mutants may lose both catalytic activity and oligomerization ability, while some other inactive PRMT mutants may still be able to contribute to cellular arginine methylation through their oligomerization interaction with and activity-enhancing effect on WT PRMTs. Furthermore, PRMT hetero-oligomerization has been increasingly recognized in recent years (48). It is possible that inactive PRMT mutants may affect methyltransferase activities of other PRMT members through hetero-oligomeric interactions. Future studies are warranted to address these important questions.

## Experimental procedures

The general chemicals and organic solvents were purchased from commercial sources at the highest purity available. All peptides were synthesized using the Fmoc solid phase synthesis method on an automated AAPPTec Focus XC synthesizer and purified on reversed phase C18 columns with the protocols described in our previous published papers (42, 45).

### Protein expression and purification

His6x-tagged human PRMT1 protein (amino acid residue 11–353) was used in the DLS and stopped-flow fluorescence assays and they were prepared as previously reported (42, 49). For cryo-EM study and biochemical assays, the His-MBP-PRMT1 (11–353) DNA sequence was cloned into modified pET28a vector by a ligation-independent method. The clones were subsequently transformed into BL21 (DE3) *Escherichia coli* RIPL cells, which were grown at 37 °C in TB medium supplemented with 100 mg/ml ampicillin until the A600 reached about 0.8. The proteins were overexpressed by the addition of IPTG to a final concentration of 0.5 mM. After an additional incubation for 8 h at 20 °C, the culture was harvested by centrifugation at 8000*g* for 20 min and stored at −80 °C. Cell pellets from a total of 4 L of culture were re-suspended in 100 ml buffer A (100 mM phosphate buffer pH 7.5, 500 mM NaCl, 10% glycerol, and 10 mM βME). The suspension was lysed by sonication and centrifuged at 35,000*g* at 4 °C and the supernatant was loaded onto a nickel affinity column pre-equilibrated with buffer A. The protein was eluted with buffer B (20 mM Hepes pH 7.5, 500 mM NaCl, and 500 mM imidazole). The fractions containing His-MBP-PRMT1 were pooled and treated with 2 mg of TEV protease per 10 mg of His-MBP-PRMT1 and incubated at 4 °C overnight (dialyzed in parallel to remove excess imidazole using buffer: 20 mM Hepes, 50 mM NaCl, and 1 mM DTT). The cleaved proteins were further separated by IMAC, and the flow-through was subsequently concentrated to 5 mg/ml and purified by SEC (Superdex 200 HR 16/60) (GE Healthcare), which was pre-equilibrated with 20 mM Hepes pH 7.5, 150 mM NaCl, and 2 mM TCEP.

### Cryo-EM sample preparation and data collection

Three molar excesses of SAH was added to purified PRMT1. The concentrated protein (0.7 mg/ml) was deposited on a glow discharged Quantifoil R1.2/1.3 holey carbon grid (Quatifoil GmbH). Excess protein was blotted away before being frozen into liquid ethane using Vitrobot Mark iV (Thermo Fisher scientific) with a temperature of 4 °C and a humidity of 100%. After vitrification, the cryo-EM grids are stored in liquid nitrogen until imaging. The data acquisition was automated using EPU-2.7.0 software (Thermo Fisher Scientific) on a 300 kV Titan Krios microscope (Thermo Fisher Scientific), equipped with a K3 Summit detector and Bio-Quantum energy filters (Gatan), operating in super-resolution mode. Raw movie stacks were recorded at a nominal magnification of 105000 × , corresponding to a pixel size of 0.83 Å/pixel (super-resolution 0.415 Å/pixel). The defocus range was set to −0.6 to −2.5 μm, and the slit width of energy filters was set to 20 eV. Each movie consisted of 40 frames with a total dose of approximately 40 e−/Å2. For PRMT1 filaments, data was acquired on a 200 kV Talos Arctica (Thermo Fisher Scientific), equipped with a Falcon III detector. Raw movie stacks were recorded at a nominal magnification of 92000 × , corresponding to a pixel size of 1.0975 Å/pixel. The defocus range was set to −1.5 to −2.5 μm, and each movie consisted of 50 frames with a total dose of approximately 50 e−/Å2. Detailed parameters for cryo-EM imaging are provided in Table S1.

### Image processing and 3D reconstruction

The super-resolution mode recorded image stacks were motion-corrected and dose-weighted using MotionCor2 (Zheng *et al.*, 2017) with a 5 × 5 patch and a two-fold binning (resulting in a pixel size of 0.83 Å/pixel). The contrast transfer function was determined from the images after motion correction and dose weighting by CTFFIND4.1 (50). All particles were semi-automated by cisTEM (51), and the selected particle coordinates were then imported into Relion 3.0 (52) for particle extraction with a box size of 384 pixels. Poor 2D class averages were removed by several rounds of 2D classification in Relion 3.0, followed by particle selection and extraction. In the final round of 2D classification, the particles in good 2D classes were transferred to cryoSPARC (Punjani *et al.*, 2017) for further 2D classification and 3D heterogeneous refinement without imposing symmetry (C1). The particles belonging to the good 3D classes were then refined into a better resolution by homogeneous with C2 symmetry. Map sharpening and resolution estimation were done in cryoSPARC. The overall resolution was estimated using the Fourier Shell Correlation = 0.143 criteria, and the local resolution was also calculated in cryoSPARC. The 3D density maps were visualized in UCSF Chimera. For helical reconstruction, the initial data process is similar to that of a single particle. After 2D classification, helical reconstruction was conducted in cryoSPRAC, with a refined twist of 103.59 degrees and a refined rise of 23.34 Å. The details of cryo-EM reconstruction are summarized in Fig. S2 and S3. The statistical information of cryo-EM reconstructions is summarized in Table S1.

### Dynamic light scattering

Indicated concentrations of PRMT1 were incubated for 10 min on ice with buffer (50 mM Hepes pH 8, 10 mM NaCl, 0.5 mM EDTA, 0.5 mM DTT) and filtered through the 0.22 μm syringe filter. For the experiment to disrupt the oligomer formation, 6 M of guanidine hydrochloride or 5 M of guanidine thiocyanate was added to the reaction. The samples were injected into the 1 ml disposable cuvette and measured by Zetasizer Nano ZS (Malvern Instruments) at 25 °C. Three measurements of 25 scans each were collected to average. The data was analyzed by the Malvern Zetasizer software v7.10.

### Mass spectrometry detection of methylated products by PRMT1

#### Methylation of R4

The reaction buffer contained 50 mM Hepes pH 8.0, 10 mM NaCl, 0.5 mM EDTA, and 0.5 mM DTT. The reaction consisted of PRMT1 at various concentrations, 10 μM of R4 (Peptide Sequence: Ac-GG**R**GGFGG**R**GGKGG**R**GGFGG**R**GGFG), and 50 μM of SAM. The reaction was at room temperature for 30 min. An equal volume of 5% TFA was added to the reaction sample for quenching. The samples were subjected to MALDI-MS analysis.

#### Methylation of PRMT1 oligomerization

For testing the processivity of the PRMT1 oligomerization, 0.5, 1, or 2 μM PRMT1-E153Q was added to the reaction. The PRMT1 concentration was kept constant at 0.5 μM. PRMT1 was incubated with PRMT1-E153Q to equilibrate for 20 min on ice. Next, 10 μM of R4 was added to the reaction mixture. SAM was added to initiate the reaction for 30 min at 25 °C in the water bath. The reaction was quenched with an equal volume of the reaction sample with 5% TFA. The data was sent to MALDI to detect methylation states. In addition to testing the R4 peptide, we tested the methylation of H4 peptide H4 (Ac-SG**R**GKGGKGLGKGGAKRHRKV) by PRMT1-WT in the presence of PRMT1-E153Q. The protocol remained the same as testing for R4, but the concentration of PRMT1-WT was 0.1 μM.

### Radioactive methyltransferase assay (filter binding assay)

#### PRMT1-WT and PRMT1 mutants concentration-dependent measurement

To perform PRMT1 and PRMT1 mutants (PRMT1-E153Q and PRMT1-H293G/E153Q) concentration-dependent measurement, each reaction contained various concentration of PRMT1 or PRMT1 mutant, 50 μM of peptide, and 50 μM SAM (48 μM cold and 2 mM ^3^H-SAM). The reaction buffer consisted of 50 mM Hepes pH 8.0, 10 mM NaCl, 0.5 mM EDTA, and 0.5 mM DTT. The reaction was incubated at room temperature for 30 min after being initiated with ^3^H-SAM SAM. An equal amount of 100% isopropanol to the total reaction volume was used to quench the reaction. The reaction liquid was transferred onto the phosphocellulose P81 filter paper (1.1 cm × 2 cm, Protein Chemistry & Metabolism Unit at St Vincent's Institute of Medical Research). The P81 filter papers containing the samples were left at room temperature for 30 min to airdry. Furthermore, the filter paper samples were washed with 50 mM NaHCO_3_ (pH 9.0) for 3 times (20 min per wash). After the wash, the filter paper samples were left to dry at room temperature overnight. The next day, each of the filter paper sample was placed in a vial with 5 ml of scintillation cocktail (Ultima Gold mixture, PerkinElmer) added. Liquid scintillation counting (counts per minute or CPM) was performed using the Beckman Coulter LS6500. The data was graphed using the GraphPad Prism 7.

#### PRMT1 mutants concentration-dependent measurement

To study the effect of PRMT1 oligomerization, 0.05 μM of PRMT1 was incubated with various concentration of PRMT1 mutant on ice for 20 min. The peptide and SAM were added to the reaction, 50 μM and 50 μM (48 μM cold and 2 mM ^3^H-SAM), respectively. The reaction was at room temperature for 30 min. The reaction was quenched with 100% isopropanol with an equal volume of the reaction. Afterward, the reaction samples were placed on the phosphocellulose P81 filter papers. The procedure was followed as in mentioned above (*PRMT1 concentration-dependent measurement*).

#### Steady-state kinetic measurement

Each reaction sample was consisted of 0.05 μM PRMT1, 15 μM ^14^C-SAM, and various concentrations of peptide. The reaction was incubated at 30 °C for 15 min and quenched with an equal volume of the reaction sample with 100% isopropanol. The samples were further transferred to the phosphocellulose P81 filter papers and followed the procedure as mentioned earlier in this section. The kinetic data was fitted to the Hill Equation using the GraphPad Prism 7 to identify the *k*_cat_, *K*_0.5_, and *n* (Hill coefficient).

(1) $$\text{Rate}\;\left(\min^{-1}\right)=\frac{v}{\left[E\right]}=\frac{k_{\text{cat}}{\left[S\right]}^{n}}{{K_{0.5}}^{n}+{\left[S\right]}^{n}}$$

### Stopped flow measurement of the dissociation rates of PRMT1-ligand complex

The trapping experiment to measure the dissociation rate of PRMT1 was carried out using the SX20 stopped-flow spectrometers (Applied Photophysics). The setting for excitation was 498 and emission was 524. A constant concentration of 0.8 μM of H4FL peptide (Peptide Sequence: Ac-SG**R**GKGGKGDpr(FL)GKGGAKRHRK, in which FL stands for fluorescein) was pre-mix with 0.5 μM, 1.5 μM, and 5.0 μM of PRMT1. The mixture was loaded on the 100 μl drive syringe. Another 2.5 ml drive syringe was loaded with reaction buffer (50 mM Hepes pH 8.0, 10 mM NaCl, 0.5 mM EDTA, and 0.5 mM DTT) to create the 1:25 dilution factor. At least three shots were averaged for each reaction and fitted to the single exponential function. F is the fluorescence intensity, t is time, A is the amplitude of fluorescence, k is the dissociation rate constant, and C is the final value of fluorescence.

(2) F=Aexp(−kt)+C

### Computational methods

#### Rosetta FlexPepDock *ab initio* docking

Using the cryo-EM structure of the hPRMT1 hexamer as a template, we isolated structures for the monomer, dimer, and tetramer. These structures served as the starting points for docking the H4 peptide using the FlexPepDock *ab initio* protocol implemented with ROSETTA (53, 54, 55). The FlexPepDock *ab initio* protocol employs a two-step approach: first, a coarse-grained representation of the protein and peptide structures is utilized to explore potential conformations of the peptide backbone across the PRMT1 oligomer surface. Subsequently, all-atom refinement, including the repacking of side chains, is performed to optimize the models. Building upon prior knowledge from the crystal structure of the PRMT1 monomer complexed with the substrate peptide (PDB ID: 1OR8) (13), we enforced constraints on the interaction between the R3 residue of the H4 peptide and two conserved glutamic acid residues, E162 and E171, of the PRMT1 while allowing flexibility for the rest of the H4 peptide. For each PRMT1 oligomer (monomer, dimer, and tetramer), a total of 100,000 docked models (decoys) were generated, which were then filtered and clustered based on their Rosetta scores and RMSD from the initial model, respectively. The primary docking configurations for each PRMT1 oligomer were chosen based on the lowest Rosetta score of the most dominant cluster.

#### Brownian Dynamics simulations

To assess how PRMT1 oligomerization impacts H4-peptide binding kinetics, we conducted Brownian Dynamics simulations using BrownDye software (56). Prior to simulation runs, we utilized the PROPKA program (57, 58) to determine the static protonation states of the proteins at physiological pH 7.0. Subsequently, PDB2PQR software (59, 60) was employed to calculate the charges and radius of each atom using the Amber force field (61). A dummy atom, devoid of charge or radius, was placed at the core of the H4 peptide to monitor its spatial positioning in relation to PRMT1. Electrostatic grids for the protein systems were generated using the Adaptive Poisson-Boltzmann Solver (APBS) (62) at an ionic strength of 50 mM. The Brownian Dynamics simulations commenced by randomly dispersing the H4 peptide around the PRMT1 oligomer, after which the substrate began to move under the influence of hydrodynamic and electrostatic forces between the molecules. Simulation termination criteria included either binding occurrence or H4 peptide escape. Binding was considered to have occurred if the dummy atom of the H4 peptide reached a spherical region within 11.75 Å (b_radius) from the PRMT1 oligomer. In each system, 250,000 trajectories (in triplicate) were initiated, with a maximum step limit of 100,000. Subsequent to the simulations, the Smoluchowski equation was applied to calculate the K_on_ (63).

#### Rosetta PRMT1-H4(1–21) peptide binding analysis

PRMT1 binding modes of H4(1–21) peptide association to PRMT1 oligomers were assessed using the Rosetta Cartesian ddG protocol (64, 65). PRMT1 monomer, dimer, and tetramer mutations were evaluated for their effect on H4(1–21) peptide binding (Fig. S8). The Rosetta FastRelax methodology was used to relax the WT structures of the PRMT1 oligomers and the H4(1–21) peptide complex. After introducing mutations, the FastRelax methodology repacks side chains within 6 Å of the mutation site. We calculate the ddG value by comparing the Rosetta score difference between the relaxed mutant and the relaxed WT-PRMT1 protein.

## Data availability

The authors confirm that the data supporting the findings of this study are available within the article and its supporting information. The models of PRMT1 monomer, dimer and tetramer with H4-peptide docked with Rosetta have been deposited in the ModelArchive database under the accession codes ma-b77jr, ma-3byf6 and ma-kvamz, respectively.

## Supporting information

This article contains supporting information.

## Conflict of interest

The authors declare that they have no conflicts of interests with the contents of this article.
