# Supplementary material for: Oligomerization of protein arginine methyltransferase 1 and its functional impact on substrate arginine methylation
Source: J Biol Chem. 2024 Nov 2;300(12):107947. doi: 10.1016/j.jbc.2024.107947 (PMC11681865; doi:10.1016/j.jbc.2024.107947)
Supplement: Supplementary Tables and Figures [file mmc1.pdf]

Supplementary Tables and Figures

Table S1. Cryo-EM data collection, refinement and validation statistics

	Tetramer (EMD- 44546) (PDB- 9BHG)	Hexamer (EMD- 44532) (PDB- 9BH4)	Octamer (EMD-44541) (PDB-9BHD)	Decamer (EMD-39814) (PDB-8Z7H)	Helix (EMD-39821) (PDB-8Z7O)
**Data collection**					
EM equipment	Titan Krios	Titan Krios	Titan Krios	Titan Krios	Talos Arctica
Voltage (kV)	300	300	300	300	200
Cs (mm)	2.7	2.7	2.7	2.7	2.7
Magnification (nominal)	105,000	105,000	105,000	105,000	92,000
Detector	K3	K3	K3	K3	Falcon III
Pixel size (Å)	0.83	0.83	0.83	0.83	1.0975
Electron exposure (e^{-}/Å^2)	~ 40	~ 40	~ 40	~ 40	~ 50
Exposure time (s)	2.5	2.5	2.5	2.5	2.5
Frames (no.)	40	40	40	40	50
Defocus range (μm)	-0.6 ~ -2.5	-0.6 ~ -2.5	-0.6 ~ -2.5	-0.6 ~ -2.5	-1.0 ~ -2.5
**Reconstruction**					
Software	Relion & cryoSPARC	Relion & cryoSPARC	Relion & cryoSPARC	Relion & cryoSPARC	Relion & cryoSPARC
Micrographs stacks (no.)	5,169	5,169	5,169	5,169	1,526
Final particle images (no.)	32,630	253,022	52,574	29,210	258,073
Symmetry imposed	C2	C2	C2	C2	Helical
Map final resolution (Å) †	3.25	2.55	3.38	3.56	3.35
Map sharpening B-factor (Å2)	-98.9	-99.9	-98.4	-103.3	-204.1
**Atomic modeling**					
Software	Coot & Phenix	Coot & Phenix	Coot & Phenix	Coot & Phenix	Coot & Phenix
Number of protein residues #	1,324	1,986	2,648	3,310	7,944
Number of ligands #	SAH: 4	SAH: 6	SAH:8	SAH: 10	SAH: 24
Number of atoms #	10,860	16,290	21,720	27,150	65,160
Map CC (around atoms) *	0.81	0.85	0.79	0.78	0.82
RMSD bond lengths (Å)	0.007	0.003	0.009	0.007	0.008
RMSD bond angles (°)	0.695	0.648	0.767	0.788	0.702
Clash score *	9.53	8.77	10.81	12.73	10.41
Ramachandran favored (%) *	96.65	98.22	96.04	97.20	95.12
Ramachandran allowed (%) *	3.35	1.78	3.96	2.80	4.88
Ramachandran outliers (%) *	0	0	0	0	0
Rotamer outliers (%) *	0	0	0	0	0
C_β deviations *	0	0	0	0	0
MolProbity score *	1.72	1.47	1.82	1.76	1.88
EMRinger score	2.30	4.64	2.68	2.15	3.41

†According to FSC=0.143; # Statistics are given for one icosahedral asymmetric unit

* According to the criterion of the reference: Chen, V. B., et al. Acta Cryst, 2010, D66, 12.

Table S2. List of calculated Rosetta ddG score rank order of PRMT1 oligomers (monomer, dimer and tetramer) across different mutations.

**Mutation**	**PRMT1_Monmer** ddG_Score	Mutation Chain_A	**PRMT1_Dimer** ddG_Score	Mutation Chain_A	**PRMT1_Tetran** ddG_Score
E217R	20.103	D69R	19.134	W312A	13.672
K228D	14.713	Y166A	10.525	K313D	7.542
D210R	12.946	K68D	6.084	Y166A	7.181
D223R	10.399	M164A	5.082	H311A	5.631
Y166A	6.492	E70R	3.485	Y57A	4.665
E65R	6.025	Y170A	3.261	M66A	4.62
Y170A	6.023	K313D	3.197	Y309A	4.484
Y57A	5.916	H311A	1.661	V239A	4.336
K313D	5.806	E65R	1.473	Y170A	4.265
V239A	4.887	P308A	1.52	E65R	2.723
M164A	4.817	Chain_B		P308A	1.803
Y208A	3.763	W215A	11.445	S56A	-0.858
Y211A	2.583	Q207A	2.577	Chain_D	
Y52A	2.56	R206D	1.879	Y208A	5.538
Q207A	1.599	D210R	1.876	V239A	4.743
D321R	1.22	Y211A	0.985	D241R	4.277
D238R	-5.47	H214A	-1.391	D205R	3.687
				P242A	2.551
				Y309A	2.20
				Y211A	1.448
				K243D	0.82
				Q207A	0.024
				Q314A	-1.90

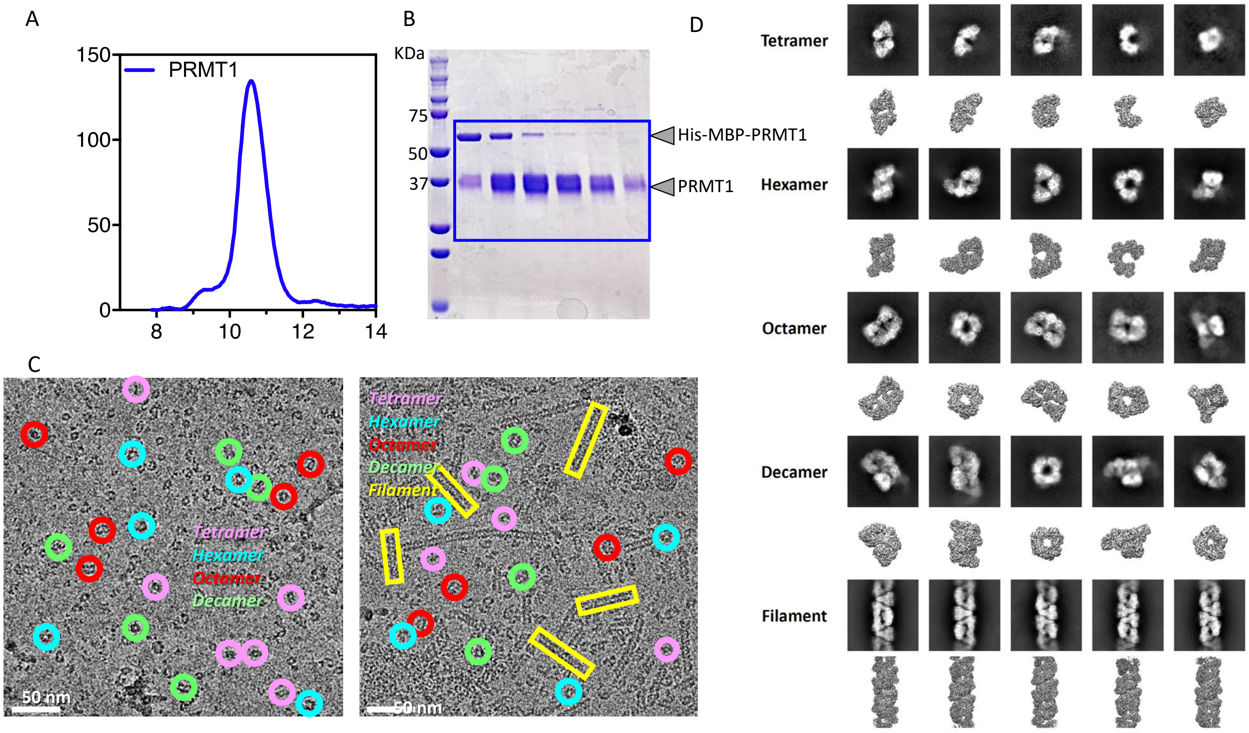

Figure S1. Structural characterization of PRMT1. (A) The SEC profile of PRMT1 is colored in blue (B) The respective samples SDS-PAGE gel highlighted in a blue box. (C) Micrographs illustrating various oligomeric states of PRMT1, Tetramer, hexamer, octamer, decomer & filament showing in pink, cyan, red, green & yellow boxes respectively. (D) 2D reconstruction of each oligomeric state of PRMT1.

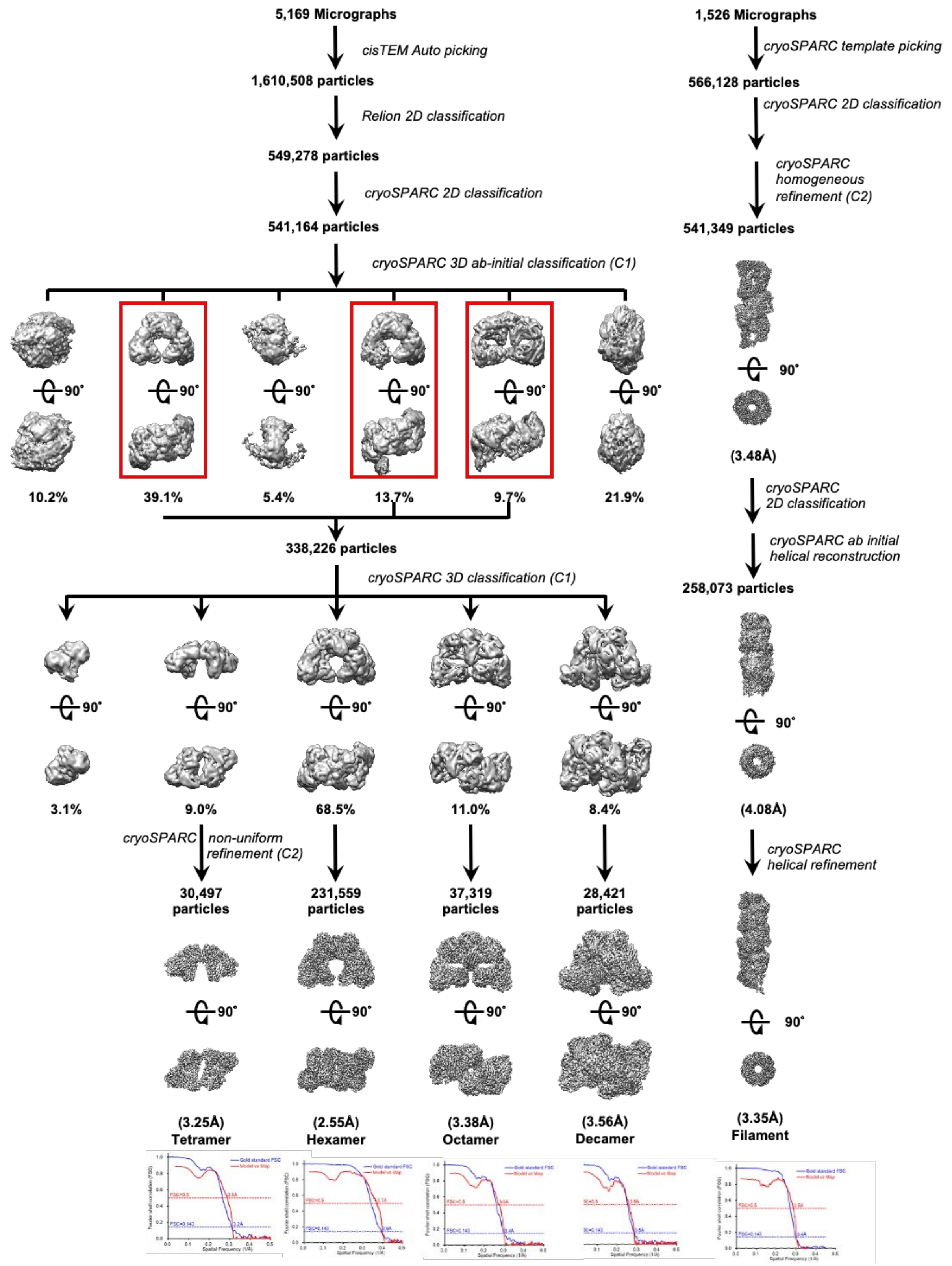

Figure S2. Workflow of the data processing of PRMT1 oligomers and filament.

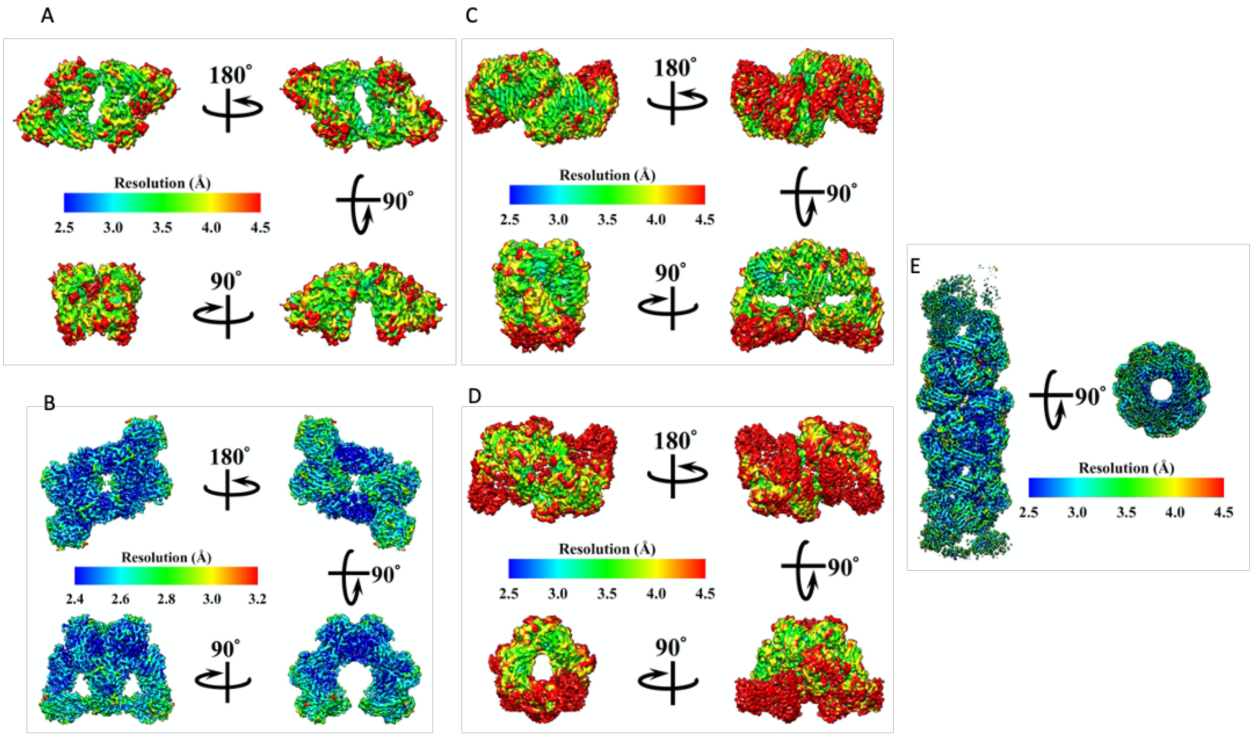

Figure S3. Local resolution estimation of (A). Tetramer (B). Hexamer (C). Octamer (D). Decomer (E). Filament.

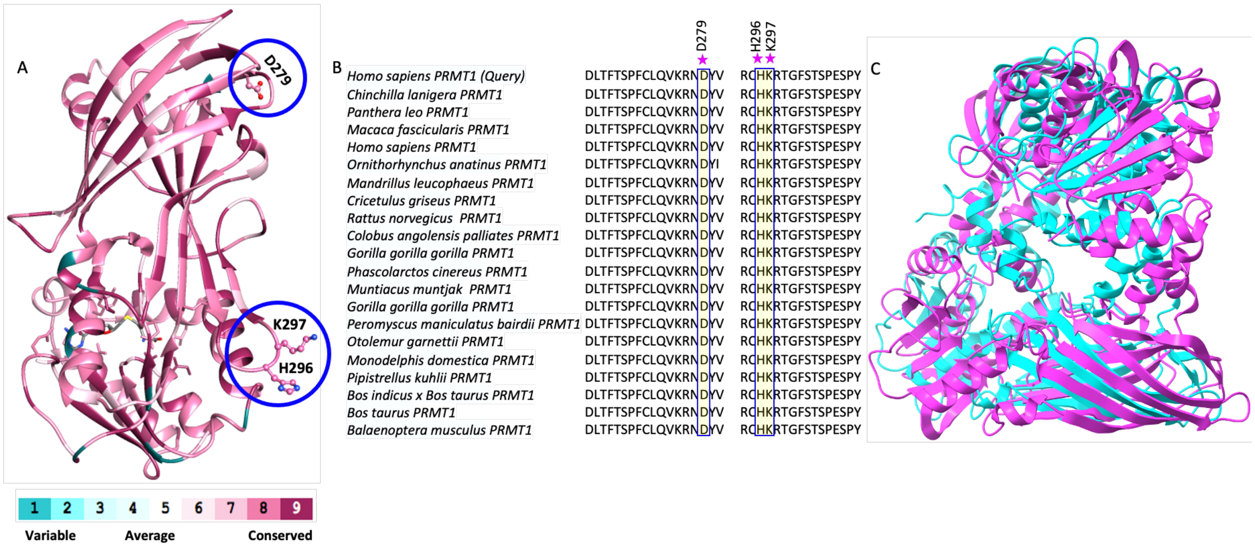

Figure S4. Evolutionary conservation of interface residues. (A) Consurf model for evolutionary conservation, the encounter residues are shown in ribbon representation with evolutionarily conserved and divergent residues colored and highlighted. Fully conserved residues are in pink and highly divergent residues are in green. The encounter interface is highlighted in blue circles. (B) Multiple sequence alignment of PRMT1 from different species. The interface residues of the PRMT1 oligomer, D279, H296 & K297 are conserved in all available species of PRMT1. (C). Superposition of PRMT8 (5DST) dimer in magenta color with PRMT1 dimer in cyan color.

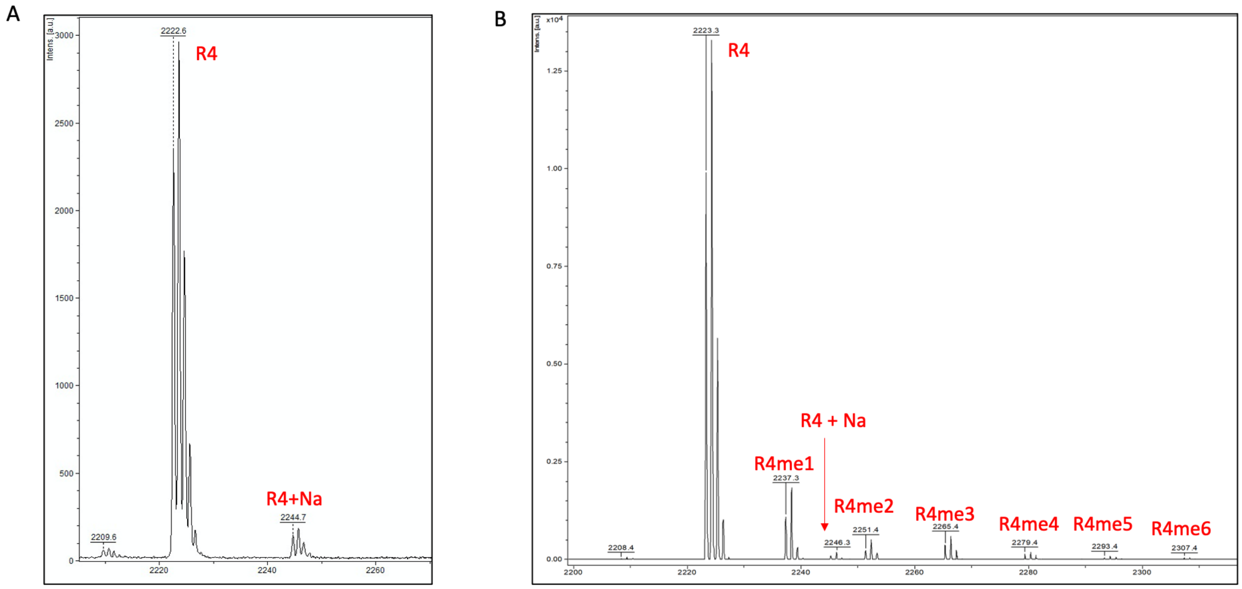

Figure S5. PRMT1 and PRMT1-E153Q activity testing using R4 peptide. The reaction consisted of 2 uM of PRMT1 or PRMT1-E153Q, 10 uM of R4 peptide, and 50 uM of SAM. The reaction was quenched with an equal amount of 5% TFA after 30 min reaction at room temperature. (A) MALDI of PRMT1-E153Q methylation activity. (B) MALDI of PRMT1 methylation activity.

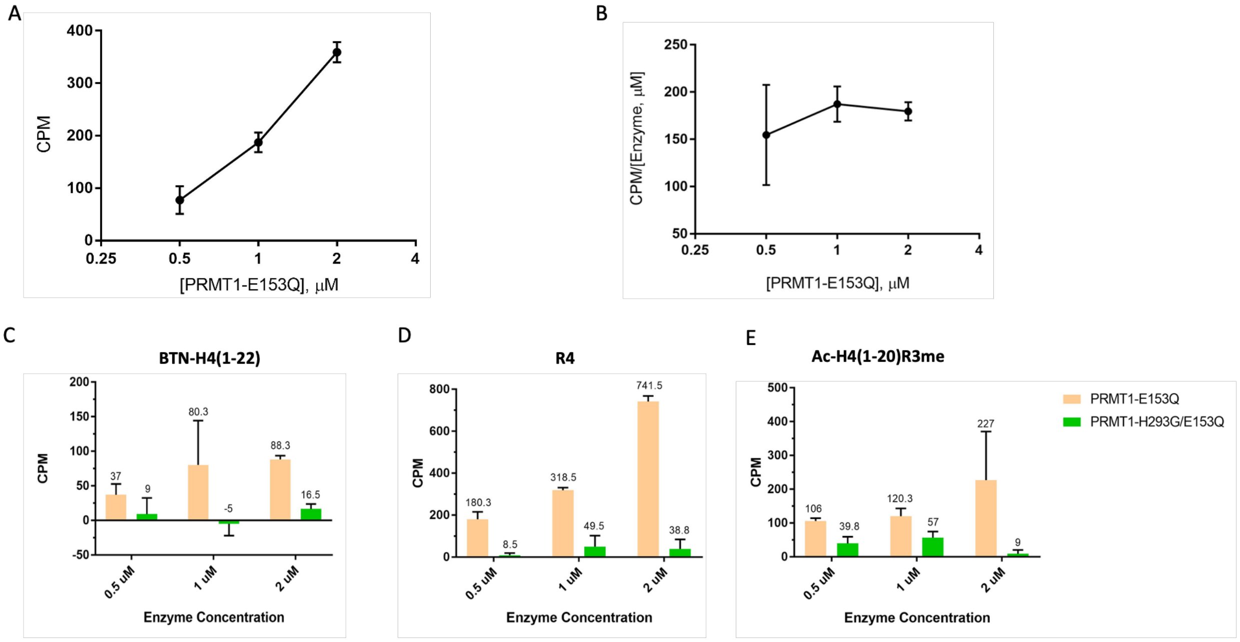

Figure S6. Determine PRMT1-E153Q and PRMT1-H293G/E153Q methyltransferase activity of R4 peptide. Various concentrations of PRMT1-E153Q or PRMT1-H293G/E153Q was incubated with radioactive 2 uM [3H]-SAM and 48 uM SAM and 50 uM of peptide for 30 min. The reaction was quenched with 100% isopropanol. (A) the methylation level of R4 peptide by PRMT1-E153Q. (B) the methylation level of R4 peptide by PRMT1 per monomer in the presence of PRMT1-E153Q. (C-E) methylation activity of PRMT1-E153Q versus PRMT1-H293G/E153Q.

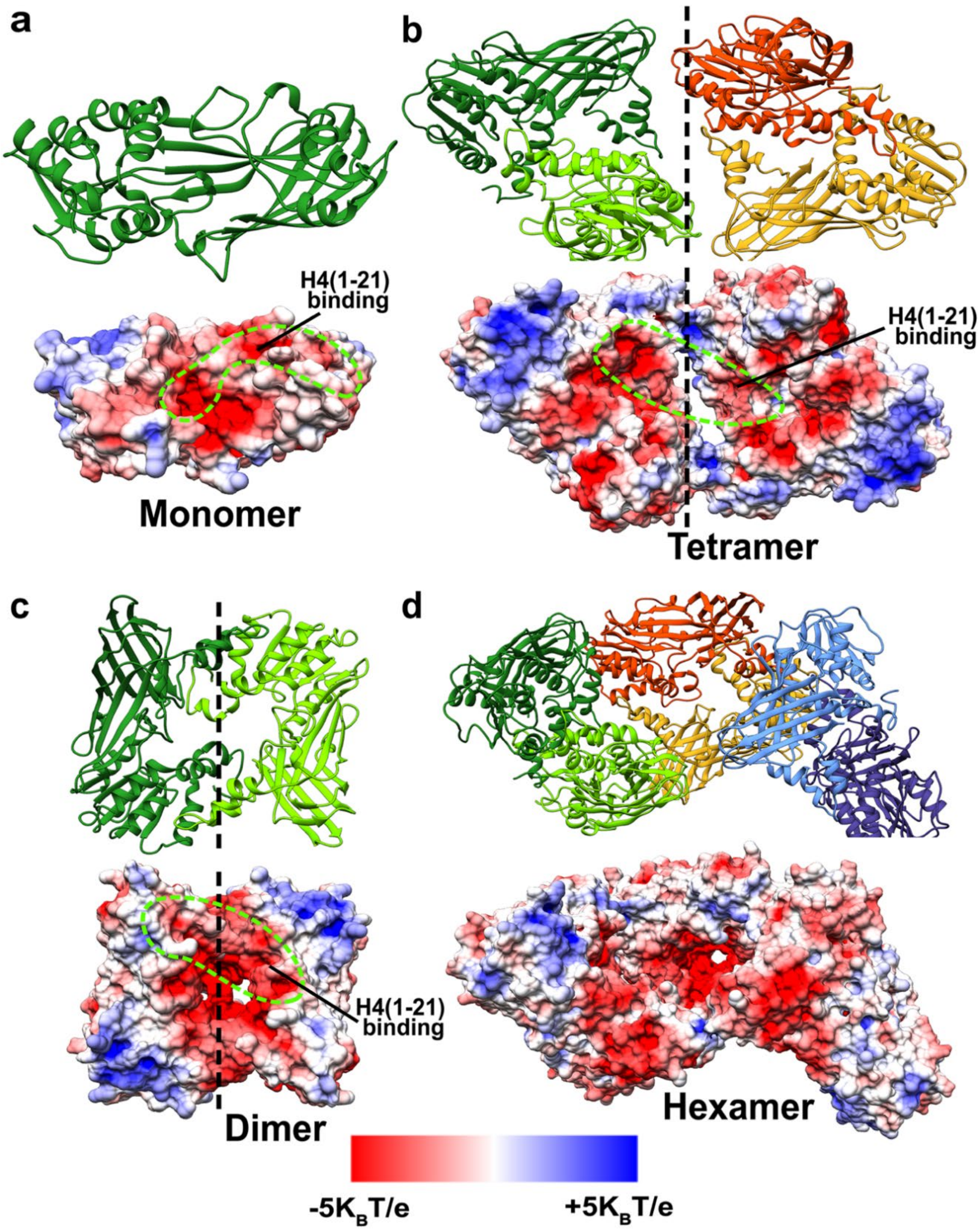

Figure S7. Structure and electrostatics of different oligomers of PRMT1. The structures are displayed alongside the electrostatic potential mapped onto the surfaces of the PRMT1 (A) monomer, (C) dimer, (B) tetramer, and (D) hexamer. In each case (except for the hexamer), the H4 peptide binding region is demarcated with a green dashed line.

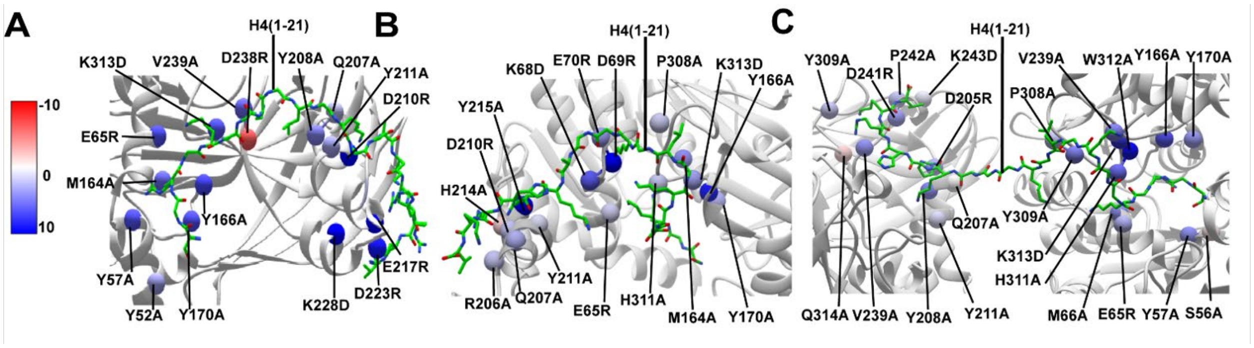

Figure S8. Mapped PRMT1 oligomers different point mutations within protein-H4(1-21) peptide interaction. (A) PRMT1 monomer, (B) PRMT1 dimer, and (C) PRMT1 tetramer mapped mutation colored by Rosetta ddG scores.
